# MiR‐192‐5p/RB1/NF‐κBp65 signaling axis promotes IL‐10 secretion during gastric cancer EMT to induce Treg cell differentiation in the tumour microenvironment

**DOI:** 10.1002/ctm2.992

**Published:** 2022-08-15

**Authors:** Jialin Song, Zaihuan Lin, Qing Liu, Sihao Huang, Lei Han, Yan Fang, Panyi Zhong, Rongzhang Dou, Zhenxian Xiang, Jinsen Zheng, Xinyao Zhang, Shuyi Wang, Bin Xiong

**Affiliations:** ^1^ Department of Gastrointestinal Surgery Zhongnan Hospital of Wuhan University Wuhan China; ^2^ Hubei Key Laboratory of Tumour Biological Behaviours Wuhan China; ^3^ Hubei Cancer Clinical Study Center Wuhan China; ^4^ Department of Respiratory and Critical Care Medicine Zhongnan Hospital of Wuhan University Wuhan China; ^5^ Wuhan Research Center for Infectious Diseases and Cancer Chinese Academy of Medical Sciences Wuhan China; ^6^ Department of obstetrics and gynecology Guangzhou Women and Children's Medical Center Guangzhou China

**Keywords:** EMT, gastric cancer, IL‐10, miR‐192‐5p, RB1, Tregs

## Abstract

**Background:**

Regulatory T (Treg) cells are important components of the tumour microenvironment (TME) that play roles in gastric cancer (GC) metastasis. Although tumour cells that undergo epithelial‐mesenchymal transition (EMT) regulate Treg cell function, their regulatory mechanism in GC remains unclear.

**Methods:**

The miR‐192‐5p was identified by examining three Gene Expression Omnibus GC miRNA expression datasets. RNA immunoprecipitation (RIP) and dual‐luciferase reporter assays were conducted to identify interactions between miR‐192‐5p and RB1. The role of miR‐192‐5p/RB1 in GC progression was evaluated based on EdU incorporation, wound healing and Transwell assays. An in vitro co‐culture assay was performed to measure the effect of miR‐192‐5p/RB1 on Treg cell differentiation. In vivo experiments were conducted to explore the role of miR‐192‐5p in GC progression and Treg cell differentiation.

**Results:**

MiR‐192‐5p was overexpressed in tumour and was associated with poor prognosis in GC. MiR‐192‐5p bound to the RB1 3′‐untranslated region, resulting in GC EMT, proliferation, migration and invasion. MiR‐192‐5p/RB1 mediated interleukin‐10 (IL‐10) secretion by regulating nuclear factor‐kappaBp65 (NF‐κBp65), affecting Treg cell differentiation. NF‐κBp65, in turn, promoted miR‐192‐5p expression and formed a positive feedback loop. Furthermore, in vivo experiments confirmed that miR‐192‐5p/RB1 promotes GC growth and Treg cell differentiation.

**Conclusion:**

Collectively, our studies indicate that miR‐192‐5p/RB1 promotes EMT of tumour cells, and the miR‐192‐5p/RB1/NF‐κBp65 signaling axis induces Treg cell differentiation by regulating IL‐10 secretion in GC. Our results suggest that targeting miR‐192‐5p/RB1/NF‐κBp65 /IL‐10 may pave the way for the development of new immune treatments for GC.

## Graphical abstract

- MiR‐192‐5p/RB1 promotes EMT of GC cells.
- EMT GC cells induce Treg cell differentiation by MiR‐192‐5p/RB1/NF‐κBp65/IL‐10 axis in the tumor microenvironment.
- MiR192‐5p/RB1 facilitates the secretion of IL‐10 by promoting the transcriptional activity of NF‐κBp65 and NF‐κBp65 in turn promotes miR‐192‐5p expression in GC.

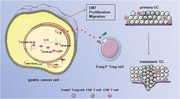

## 1 INTRODUCTION

Gastric cancer (GC) is one of the most prevalent tumours with a high mortality rate, particularly in China.^1^, ^2^ Metastasis is the mean cause of death in GC patients. The tumour microenvironment (TME), which consists of non‐malignant cells, immune cells and the inflammatory mediators they secrete,^3^ plays a vital role in tumour metastasis.^4^, ^5^ Regulatory T cells (Tregs), as a subtype of CD4^+^ T cells, accumulate in the TME and play vital roles in tumour metastasis.^6^ Large populations of FOXP3^+^ Tregs have been recognized in the TME, and their accumulation has been linked to poor prognosis in cancer.^7^, ^8^ Elevated FOXP3^+^ Tregs have been linked to poor overall survival and tumour metastasis in GC.^9^, ^10^ Therefore, investigation of the potential mechanisms underlying Treg cell and cancer cell interaction is essential for understanding the mechanisms of metastatic process in GC.

Epithelial–mesenchymal transition (EMT) is crucial for tumour metastasis.^11^ Tumour cells undergoing EMT exhibit mesenchymal phenotypes that promote cell migration. EMT promotes tumour metastasis by modulating the TME,^12^, ^13^ it induces an immunosuppressive TME, which confers immune escape, and metastasis in tumour cells. During tumour EMT, tumour cells secret cytokines including TGF‐β and interleukin‐10 (IL‐10) to induce T cells into Tregs,^14^, ^15^ thereby promoting tumour progression and metastasis.^16^, ^17^ Additionally, TGF‐β‐induced Tregs have been shown to promote tumour metastasis in B16‐F10 mouse.^18^ However, the specific mechanisms by which EMT tumour cells promote Treg cell differentiation in GC have not yet been evaluated.

MicroRNA (miRNA) is small non‐coding RNA, 17‐25‐nucleotide in lengths, that can bind to target mRNA resulting in mRNA translational inhibition or degradation.^19^ MiRNAs are dysregulated in tumour cells undergoing EMT.^20^, ^21^ In GC, miRNAs promote tumour metastasis by modulating the tumour cell EMT.^22^, ^23^ MiRNAs in tumour cells promote Treg cell differentiation, thereby affecting tumour metastasis.^24^, ^25^ Whereas the potential mechanisms of miRNA dysregulation induce tumour cells to promote Treg cell differentiation remains largely unknown. Given the importance of miRNA in tumour EMT and Tregs, we speculate that miRNA induces EMT of tumour cells and secretes regulatory molecules to induce Treg cell differentiation.

RB1, as a tumour suppressor, plays important role in cell cycle and metastasis in many cancers.^26^, ^27^ The altered RB1 affected the production of cytokines and chemokines in tumour cells.^28^ Through bioinformatics analysis, we found that RB1 may be a target of miR‐192‐5p. Since we unveiled that miR‐192‐5p promotes tumour progression and metastasis in GC. We investigated the possibility that miR‐192‐5p/RB1 could act as a mediator in the TME during GC EMT. Our findings might offer insights on how miRNA dysregulation induces tumour cells to promote Treg cell differentiation and facilitate the development of new immunotherapies for GC.

## 2 RESULTS

### 2.1 MiR‐192‐5p is overexpressed in GC and associated with RB1 expression

**FIGURE 1:**
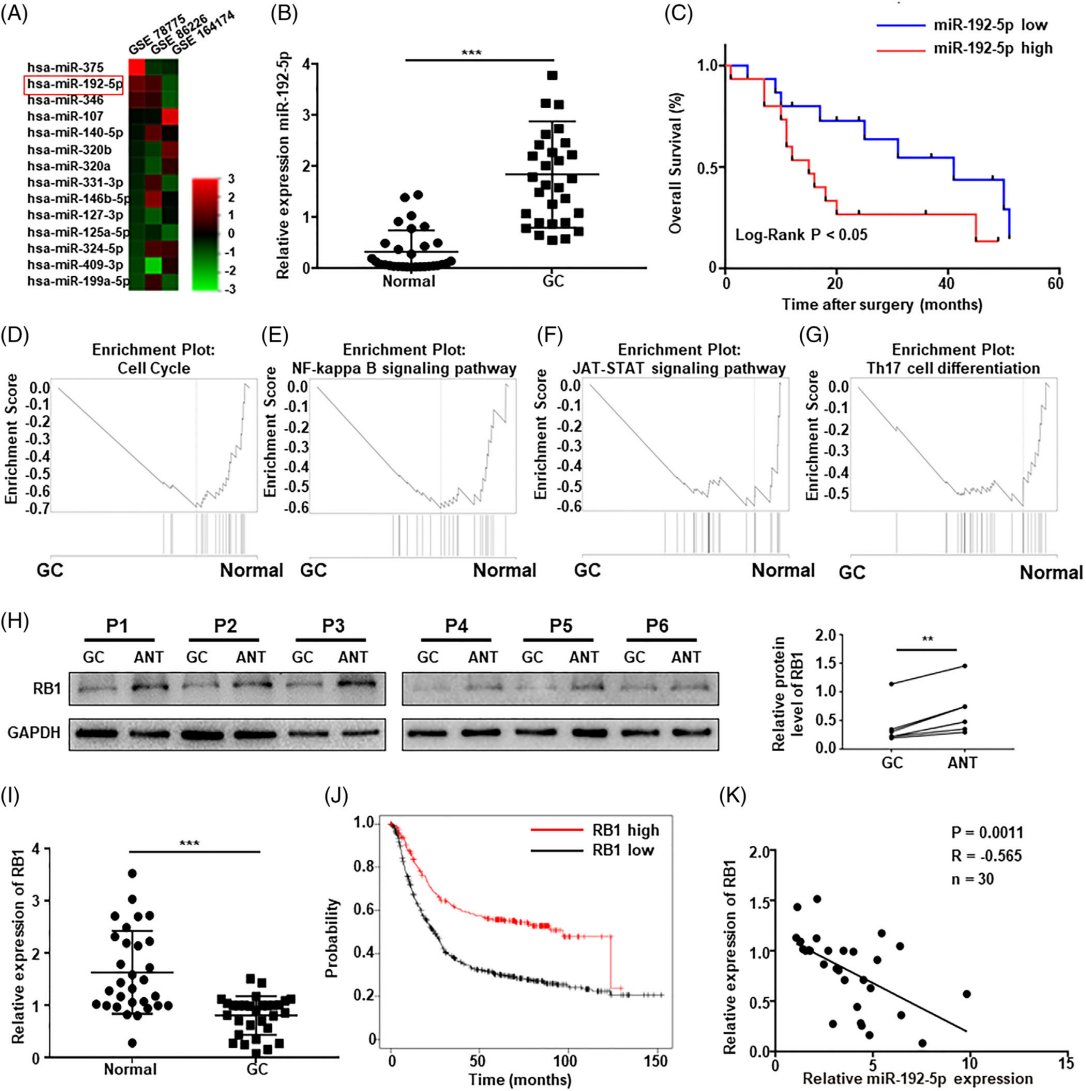
miR‐192‐5p was overexpressed in gastric cancer (GC) tissues and associated with low expression of RB1. (A) Differentially expressed miRNAs in GC based on gene expression omnibus (**GEO)** database data. (B) The expression of miR‐192–5p was examined in 30 pairs of GC and adjacent normal tissue (ANT) by qRT‐PCR. U6 was used for qRT‐PCR normalization of miR‐192–5p. (C) The 30 GC samples were divided into two groups based on the median expression of miR‐192–5p, then the Kaplan–Meier survival analysis was conducted to examine the overall survival rate. (D–F) Gene set enrichment analysis (GSEA) analysis of target genes for miR‐192‐5p. (H) The protein levels of RB1 in 6 pairs of GC samples. (I) RB1 expression in 30 paired of GC and ANT. Glyceraldehyde phosphate dehydrogenase (GAPDH) was used for qRT‐PCR normalization of RB1. (J) The overall survival curves of 875 GC patients with different RB1 expression on the Kaplan–Meier Plotter website (http://kmplot.com/analysis). (K) The correlation between RB1 and miR‐192‐5p was analyzed. Statistical analysis between two groups was conducted using two‐tailed *t*‐test. Error bars, standard deviation (SD). *p < .05, **p < .01, ***p < .001

Based on the gene expression omnibus (GEO) database (accession numbers: GSE78775, GSE86226 and GSE164174), we measured miRNA expression in 1454 GC and normal samples (Figure 1A). We identified 14 miRNAs that were in all three datasets, among which, miR‐192‐5p expression was elevated in tumours. Subsequently, we collected 30 pairs of GC and adjacent normal tissue (ANT) samples to assess miR‐192‐5p expression (Table S1). Quantitative real‐time polymerase chain reaction (qRT‐PCR) result demonstrated that miR‐192‐5p expression was considerably elevated in GC compared to ANT (Figure 1B, Figure S1G, *p* < .001). Additionally, miR‐192‐5p overexpression was associated with N stage (Table S1, *p* = .021) and showed a short survival rate (Figure 1C). MiR‐192‐5p was an independent prognostic factor for GC patients (Table S3).

Target prediction tools (TargetScan, Microrna, Starbase, and miRWalk) were utilized to explore target gene of miR‐192‐5p; gene set enrichment analysis (GSEA) was performed on predicted genes (Figure 1D–G). Interestingly, the predicted genes were enriched in cell cycle and EMT pathways such as nuclear factor‐kappaB (NF‐κB) pathway, JAT‐STAT pathway, PI3K‐Art signaling pathway and Th17 cell differentiation. Among the predicted genes, the predicted gene RB1, as a tumour suppressor that regulates the cell cycle, is known to participate in immune cell infiltration and multiple signaling pathways, such as the NF‐κB signaling pathway.^30^ Based on these findings, we further investigated the relationship between RB1 and miR‐192‐5p. We measured RB1 expression in 30 pairs of GC and ANT samples (Figure 1H,I, Figure S1H). RB1 expression was substantially lower in GC (Figure 1I, *p* < .001), and decreased RB1 expression was vastly associated with poor overall survival (OS) in GC (Figure 1J, *p* < .001). In addition, miR‐192‐5p expression was inversely linked with RB1 expression (Figure 1K, *p* = .0011).

### 2.2 RB1 is targeted and inhibited by miR‐192‐5p

**FIGURE 2:**
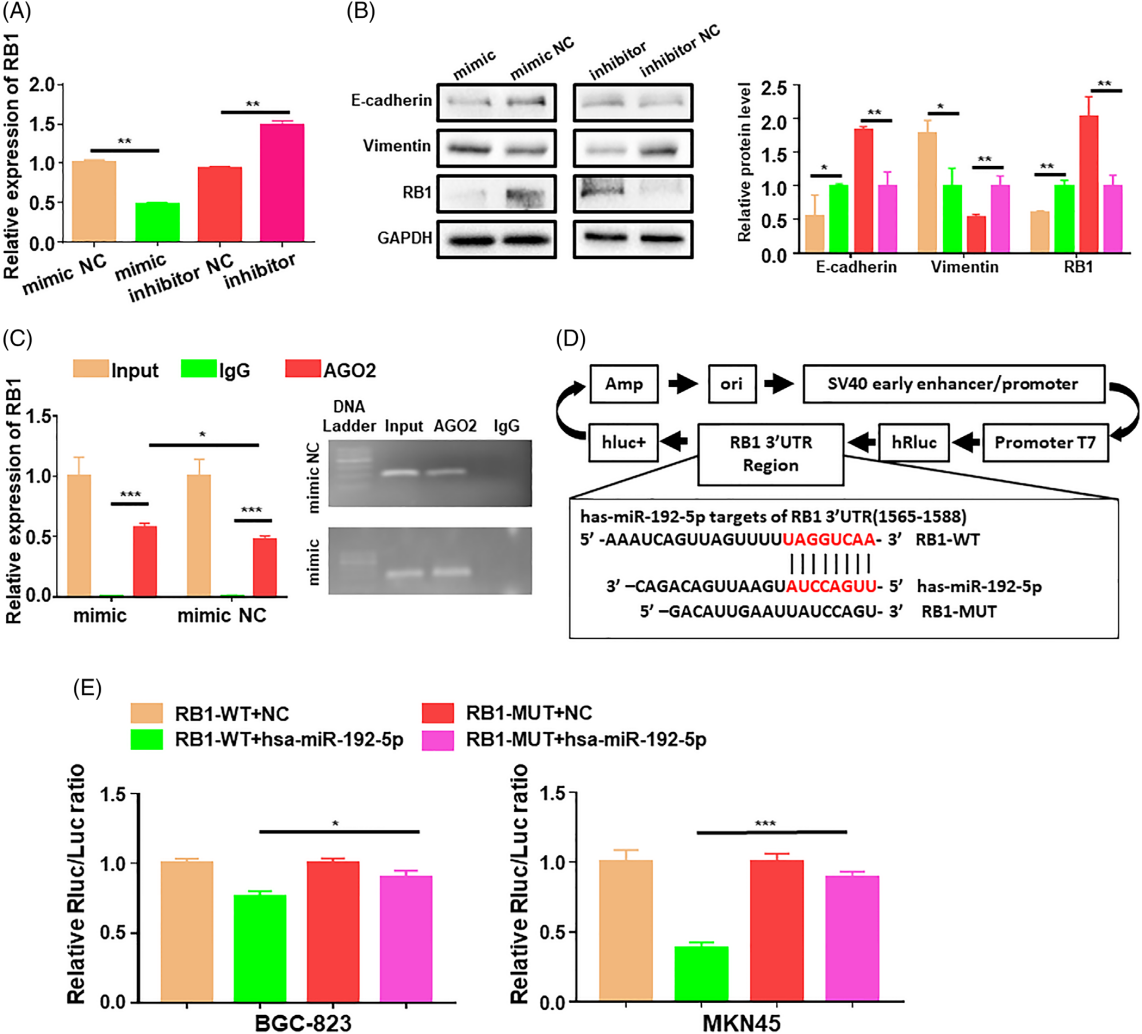
RB1 is a direct target of miR‐192‐5p in gastric cancer (GC). (A) The RB1 mRNA level in transfected GC cells was determined by qRT‐PCR. GAPDH was used for qRT‐PCR normalization of RB1. (B) Western blots and WB quantification of E‐cadherin, Vimentin, RB1 in GC cells transfected with miR‐192‐5p mimic or inhibitor. (C) RNA immunoprecipitation (RIP) assay was used to detect the combination of RB1 and miR‐192‐5p. (D) Schematic graph of RB1 3′‐UTR with the putative binding sites of miR‐192‐5p. (E) The luciferase activities of BGC‐823 cells or MKN45 cells co‐transfected with miR‐192‐5p and luciferase vectors containing RB1 3′‐UTR WT or RB1 3′‐UTR MUT were determined by dual‐luciferase reporter assay. Data are pooled from three independent experiments. Statistical analysis between two groups was conducted using two‐tailed *t*‐test. One‐way analysis of variance (ANOVA) statistical tests were adopted for more than two groups. Error bars, standard deviation (SD). *p < .05, **p < .01, ***p < .001

We then probed the mechanism via which miR‐192‐5p regulates RB1. MiR‐192‐5p expression was evaluated in multiple cell lines (Figure S4A), which was overexpressed in GC cells, then MKN45 and BGC‐823 cells were selected as the experimental cell lines for subsequent experiment. GC cells were transfected with miR‐192‐5p mimic or inhibitor to overexpress or knockdown miR‐192‐5p, respectively. Changes in RB1 expression were then detected, and miR‐192‐5p markedly altered RB1 expression in GC cells (Figure 2A,B). In addition, miR‐192‐5p overexpression significantly suppressed E‐cadherin expression (*p* = .041) and increased vimentin expression (*p* = .015) in GC cells. While increased E‐cadherin (*p* = .002) and declined vimentin expression (*p* = .006) were detected in miR‐192‐5p inhibitor cells (Figure 2B).

To further prove that RB1 is the target of miR‐192‐5p, RNA immunoprecipitation (RIP) was performed using anti‐Ago2 or control IgG, and the immunoprecipitates were analyzed by PCR (Figure 2C). RIP results showed that RB1 was significantly enriched in Ago2‐coated beads (*p* < .001). In addition, RB1 enrichment was up‐regulated in MKN45 cells transfected with miR‐192‐5p compared to that in cells transfected with mimic negative control (NC) (*p* = .0157). Furthermore, dual‐luciferase reporter assay was carried out by cloning RB1‐3′‐untranslated region (UTR) into the luciferase reporter plasmid. Sequence alignment showed that RB1 contains a miR‐192‐5p binding site (Figure 2D). The luciferase activities were significantly decreased in GC cells transfected with the wide tyle (WT)‐3′ UTR‐RB1 and the miR‐192‐5p (*p* = .0220), while it was not significantly altered when GC cells were transfected with the mutant type (MUT)‐3′ UTR‐RB1 and the miR‐192‐5p (Figure 2E).

### 2.3 MiR‐192‐5p/RB1 promotes GC cell proliferation, migration and invasion

**FIGURE 3:**
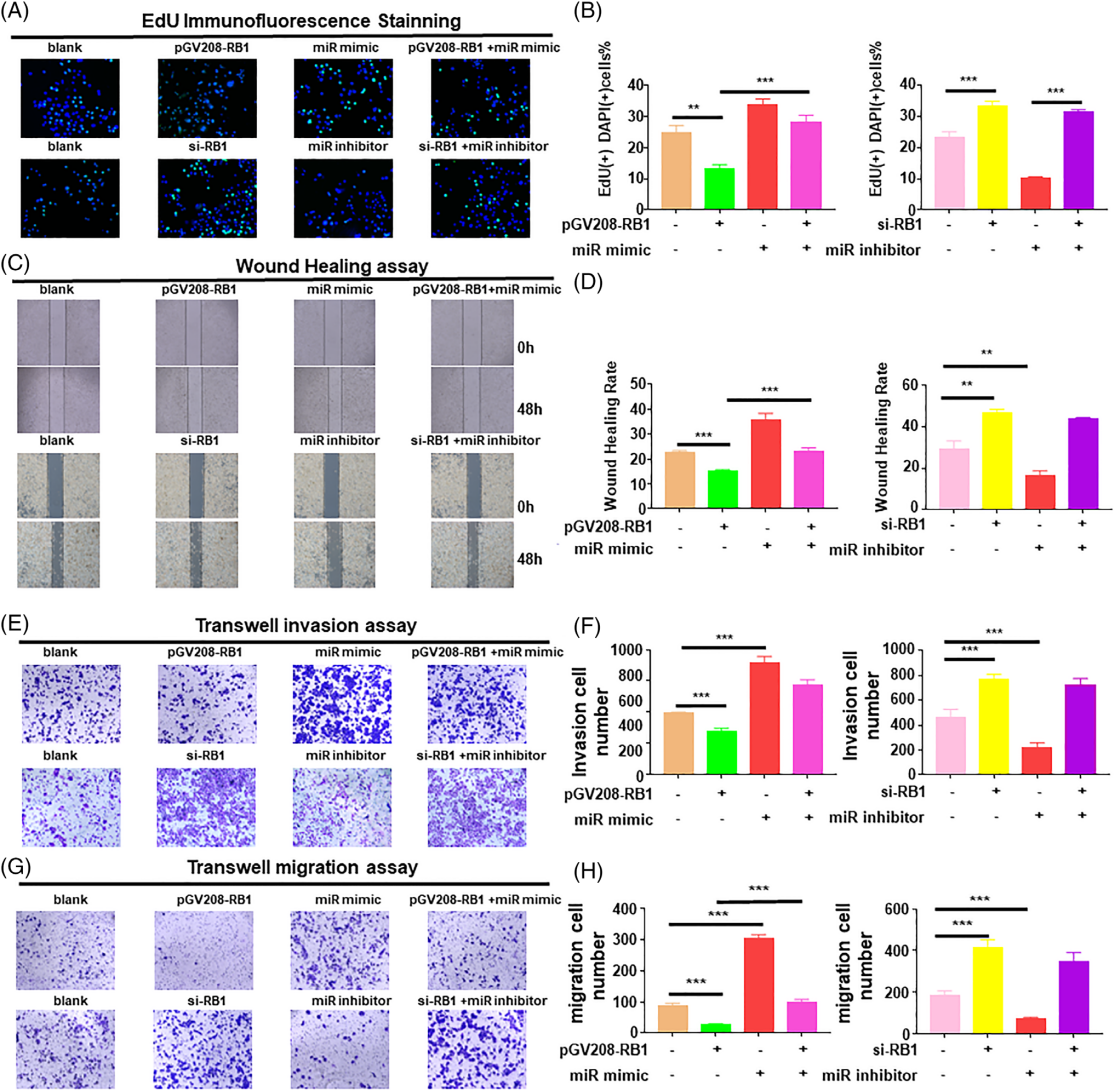
MiR‐192‐5p regulated RB1 to affect proliferation, migration, invasion of gastric cancer (GC) cells in vitro. BGC‐823 cells were co‐transfected with miR‐192‐5p mimic and oe‐RB1; MKN45 cells were co‐transfected with miR‐192‐5p inhibitor and si‐RB1. The GC cells were stained by crystal violet to evaluate cell migration and invasion capability. (A) Cell proliferation abilities were determined by EdU staining. Scale bar, 50 μm. (B) Quantifications of EdU staining assay. (C) Cell migration abilities were checked by wound healing assay of transfected GC cells. Scale bar, 100 μm. (D) Quantifications of wound healing assay. (E) Migration of transfected GC cells was measured by Transwell migration assays. Scale bar, 100 μm. (F) Total number of migratory cells in five fields was counted manually. (G) Cell invasion capability was measured by transwell assay. Scale bar, 100 μm. (H) Total number of invasive cells in five fields was counted manually. Data are pooled from three independent experiments. One‐way ANOVA statistical tests were adopted for more than two groups. Error bars, standard deviation (SD). *p < .05, **p < .01, ***p < .001

Next, we explored the roles of miR‐192‐5p/RB1 in GC cell progression. EdU immunofluorescence (IF) staining (Figure 3A and B) confirmed that miR‐192‐5p promoted cell proliferation (*p* = .0086), whereas RB1 suppressed cell proliferation in BGC‐823 cells (*p* = .0022). Moreover, RB1 up‐regulation abolished the miR‐192‐5p overexpression‐mediated BGC‐823 cell proliferation (*p* = .0006). Conversely, the miR‐192‐5p inhibitor restrained MKN45 cell proliferation (*p* < .001). In addition, RB1 depletion facilitated GC cell proliferation (*p* < .001), whereas miR‐192‐5p inhibitor could not restore this effect (*p* = .0591).

We then explored the effects of miR‐192‐5p/RB1 on cell migration and invasion. In the wound healing assay, miR‐192‐5p‐overexpressed cells showed stronger migration abilities at 48 h than NC (*p* = .0013), while miR‐192‐5p–decreased cells showed reduced migration abilities (*p* = .0082). Similar results were seen in GC cells upon manipulating RB1 expression levels (Figure 3C,D). The Transwell migration assay results were in accordance with the results of the wound healing assay (Figure 3E,F), miR‐192‐5p notably stimulated tumour cell invasion, miR‐192‐5p inhibitor restrained tumour cell invasion (Figure 3G,H). Up‐regulated RB1 abrogated miR‐192‐5p overexpression‐induced migration and invasion in BGC‐823 cells (*p* < .001). However, RB1‐down‐regulation induced MKN45 cell migration, which was not restrained by the miR‐192‐5p inhibitor (*p* = .119). Given all this, our findings reveal that miR‐192‐5p promotes GC cell progression by regulating RB1.

### 2.4 MiR‐192‐5p/RB1 promotes Treg cell differentiation in the GC TME

**FIGURE 4:**
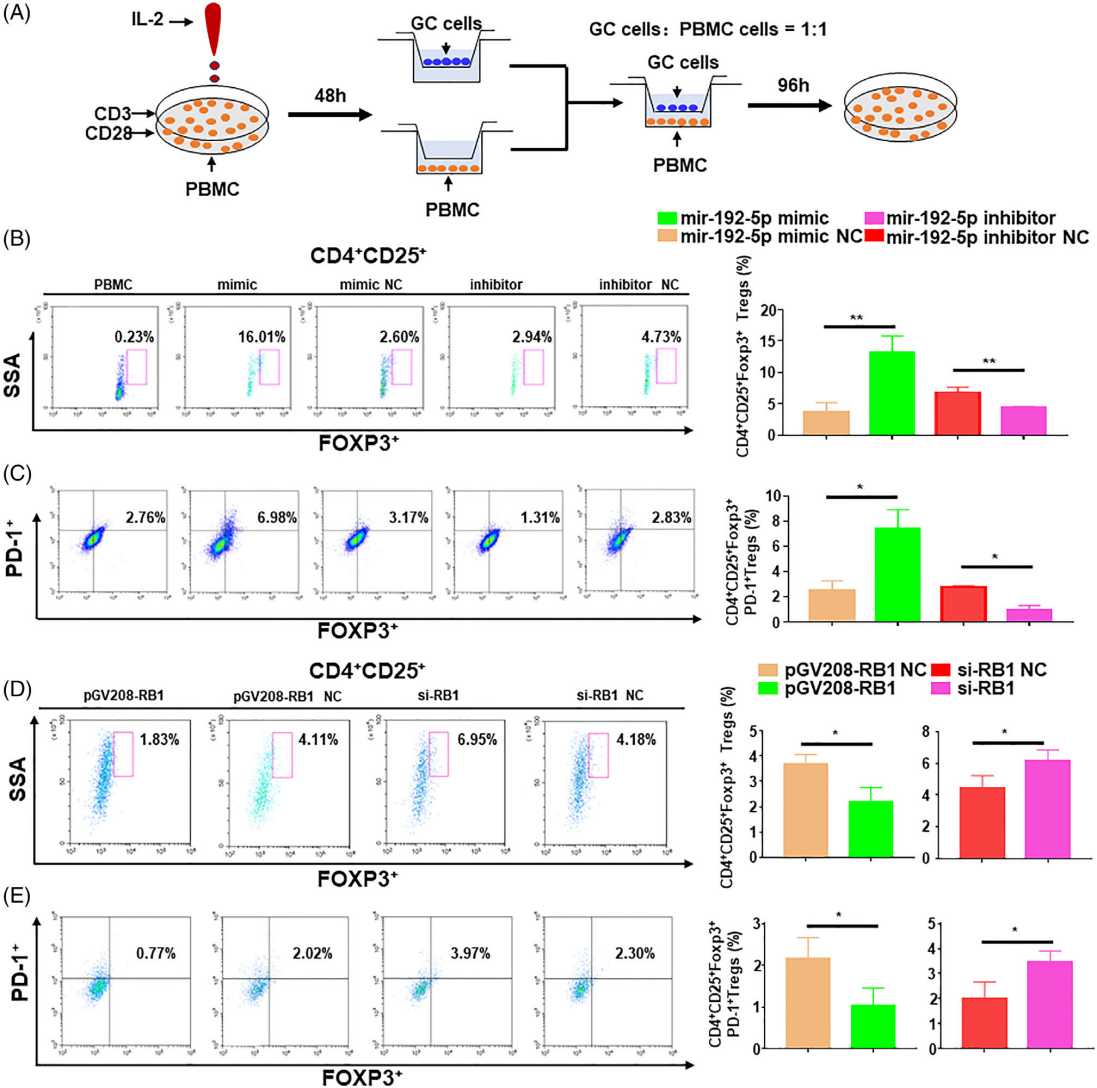
MiR‐192‐5p regulated RB1 to induce the Treg cell differentiation in vitro. (A) Peripheral blood mononuclear cells (PBMCs) were co‐cultured with transfected BGC‐823 cells or MKN45 cells. (B) Flow cytometric of CD4^+^CD25^+^FOXP3^+^ Tregs after co‐cultured with transfected gastric cancer (GC) cells. (C) Flow cytometric measured the infiltration of CD4^+^CD25^+^FOXP3^+^PD‐1^+^ Tregs. Assays were performed in triplicates. (D) Flow cytometric of CD4^+^CD25^+^FOXP3^+^ Tregs after being co‐cultured with GC cells transfected with pGV208‐RB1/pGV208‐RB1 NC/si‐RB1/si‐RB1 NC. (E) Flow cytometric measured the infiltration of CD4^+^CD25^+^FOXP3^+^PD‐1^+^ Tregs after being co‐cultured with transfected GC cells. Data are pooled from three independent experiments. Statistical analysis between two groups was conducted using two‐tailed *t*‐test. One‐way ANOVA statistical tests were adopted for more than two groups. Error bars, standard deviation (SD). *p < .05, **p < .01, ***p < .001

EMT in tumour induces immunosuppressive cells such as tumour‐associated macrophage and Tregs by expressing cytokines, thereby shaping the TME to promote cancer metastasis.^31^, ^32^ We investigated whether miR‐192‐5p/RB1 induces Treg cell differentiation in the GC TME by performing bioinformatics analysis, and result revealed that RB1 was negatively correlated with the T helper cell 17 (Th17) differentiation pathway, which contains differentiation of TH17 and Tregs, respectively. We then investigated the relevance between miR‐192‐5p/RB1 and the genes representing Th17 cells and Tregs. The results showed FOXP3 was expressed at a significant level in GC (Figure S1B,H), while IL17A expression was not different between GC and ANT samples (Figure S1A,H). IF staining showed that tumour tissues exhibited almost no positive CD4IL17A expression. However, there was significantly positive CD4FOXP3 expression in tumour tissues (Figure S2A). Furthermore, miR‐192‐5p and RB1 expression levels were markedly correlated with FOXP3 but not with IL17A (Figure S1C–F). The data suggest that miR‐192‐5p/RB1 is relevant to Tregs but not Th17 cells in GC. Therefore, we examined whether miR‐192‐5p promotes Treg cell differentiation by inhibiting RB1. Since Programmed cell death protein 1 (PD‐1) was a characteristic protein of Tregs, which could enhance the Tregs immunosuppressive function,^33^, ^34^ IF results demonstrated that tumour tissues expressed high expression of PD‐1 (Figure S3C), we detected PD‐1 expression on Tregs. A GC cells and peripheral blood mononuclear cells (PBMCs) co‐culture system was established (Figure 4A). GC cells transfected with miR‐192‐5p or RB1 were co‐cultured with activated PBMCs in a ratio of 1:1(2 × 105 cells/ml) (Figure S7A,B). After 96 h of co‐cultivation, the ratios of FOXP3^+^ and PD‐1^+^ Tregs were measured by flow cytometry. When miR‐192‐5p mimic‐transfected GC cells were co‐cultured with PBMCs, the ratios of FOXP3^+^ Tregs and PD‐1^+^Tregs were higher than those in the NC (Figure 4B,C, *p* = .0053). Besides, similarly to the miR‐192‐5p overexpression, RB1 knockdown elevated the proportions of Tregs (Figure 4C,D, *p* = .0451). Conversely, miR‐192‐5p inhibitor (*p* = .0078) or pGV208‐RB1 (*p* = .0221) decreased the proportions of FOXP3^+^ and PD‐1^+^ Tregs significantly (Figure 4E). Overall, the results indicated that miR‐192‐5p/RB1 induces Tregs differentiation in GC.

### 2.5 MiR‐192‐5p/RB1 induces Treg cell differentiation by regulating IL‐10 secretion in GC

**FIGURE 5:**
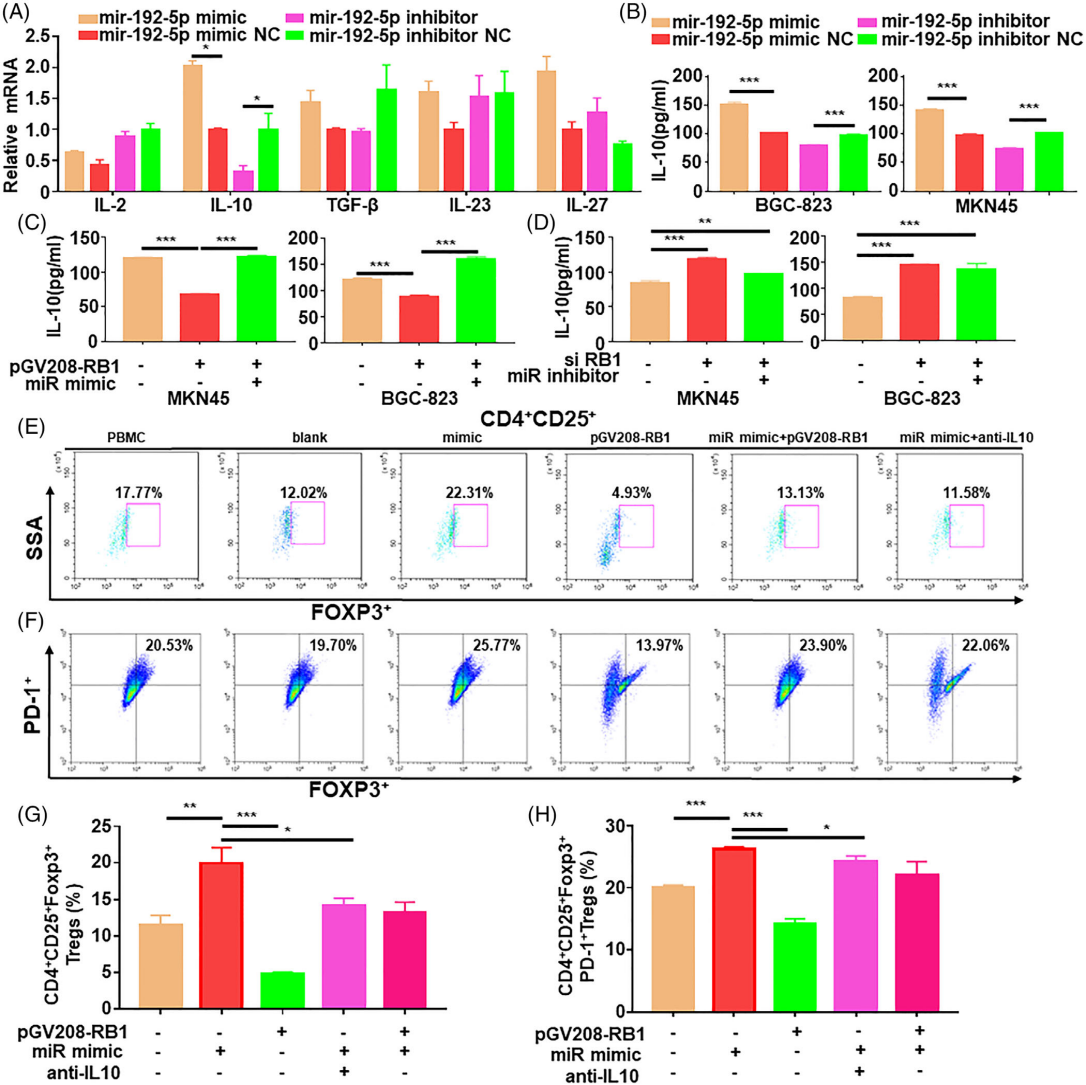
MiR‐192‐5p regulated RB1 to affect the secretion of IL‐10, inducing the Treg cell differentiation Tregs in vitro. (A) qRT‐PCR tested the cytokines that induces Treg cell differentiation in BGC‐823 cells transfected with miR‐192‐5p mimic or inhibitor. GAPDH was used for qRT‐PCR normalization of mRNA. Standard deviation (SD) (B) ELISA detected the IL‐10 levels in supernatants of GC cells transfected with miR‐192‐5p mimic/mimic NC/inhibitor/inhibitor NC. (C) The secretion of IL‐10 was detected by ELISA. BGC‐823 cells and MKN45 cells were transfected with pGV208‐RB1, miR‐192‐5p mimic. (D) ELISA detected the IL‐10 levels in supernatants of BGC‐823 cells and MKN45 cells transfected with si‐RB1, miR‐192‐5p inhibitor. (E) The infiltration of FOXP3^+^Tregs was measured by flow cytometric after being co‐cultured with transfected BGC‐823 cells. (F) Flow cytometric detected the infiltration of PD‐1^+^Tregs after being co‐cultured with transfected BGC‐823 cells. (G) Quantification of FOXP3^+^ expression on Tregs. (H) Quantification of PD‐1^+^ expression on Tregs. Data are pooled from three independent experiments. Statistical analysis between two groups was conducted using two‐tailed *t*‐test. One‐way ANOVA statistical tests were adopted for more than two groups. Error bars, standard deviation (SD). *p < .05, **p < .01, ***p < .001

Based on the above results, we next sought out to illustrate the specific mechanism via which miR‐192‐5p/RB1 regulates Treg cell differentiation. First, we analyzed several cytokines expressions that were related to Treg cell differentiation. The results showed that IL‐10 expression was notably increased in the miR‐192‐5p overexpressed cells (*p* = .0132), while profoundly decreased in the miR‐192‐5p depleted cells (*p* = .0128) (Figure 5A). Then we detected secreted IL‐10 levels in GC cell supernatant by ELISA. IL‐10 secretion was markedly increased in the miR‐192‐5p overexpressed cells (*p* < .001), decreased in the miR‐192‐5p‐deficient cells (*p* < .001) (Figure 5B). MiR‐192‐5p reversed the decreased IL‐10 level induced by RB1 in GC cells (*p* < .001), while miR‐192‐5p depletion has little impacts on the secretion of IL‐10 in RB1 knockdown GC cells (*p* = .2317) (Figure 5C,D). Collectively, the data demonstrated that miR‐192‐5p/RB1 could regulate IL‐10 secretion in GC cells. In addition, ELISA and immunohistochemistry results showed that IL‐10 levels were significantly elevated GC (Figure S5A and B).

To verify that miR‐192‐5p/RB1 promotes the Treg cell differentiation by IL‐10 secretion, we co‐cultured PBMCs with tumour cells under different conditions. MiR‐192‐5p improved the ratios of FOXP3^+^ and PD‐1^+^ Tregs, which were depressed by RB1 (Figure 5E–G). IL‐10 neutralizing antibodies suppressed the miR‐192‐5p‐induced Treg cell differentiation and PD‐1 expression (*p* = .0104) (Figure 5F–H). IL‐10 recombinant proteins significantly promoted the Tregs differentiation, which was inhibited by miR‐192‐5p inhibitor (*p* = .0012) (Figure S8A,B). In addition, miR‐192‐5p/RB1 regulated programmed death ligand1 (PDL1) (Figure S3A), which implied that miR‐192‐5p/RB1 might promote immune evasion and immune surveillance by regulating PDL1. IF results revealed high PDL1 expression in the GC compared with ANT (Figure S3B). The findings indicated that in the EMT GC cells, miR‐192‐5p/RB1 axis promotes Treg cell differentiation via IL‐10 secretion.

### 2.6 MiR‐192‐5p/RB1 induces the secretion of IL‐10 via the NF‐κBp65 signaling pathway

**FIGURE 6:**
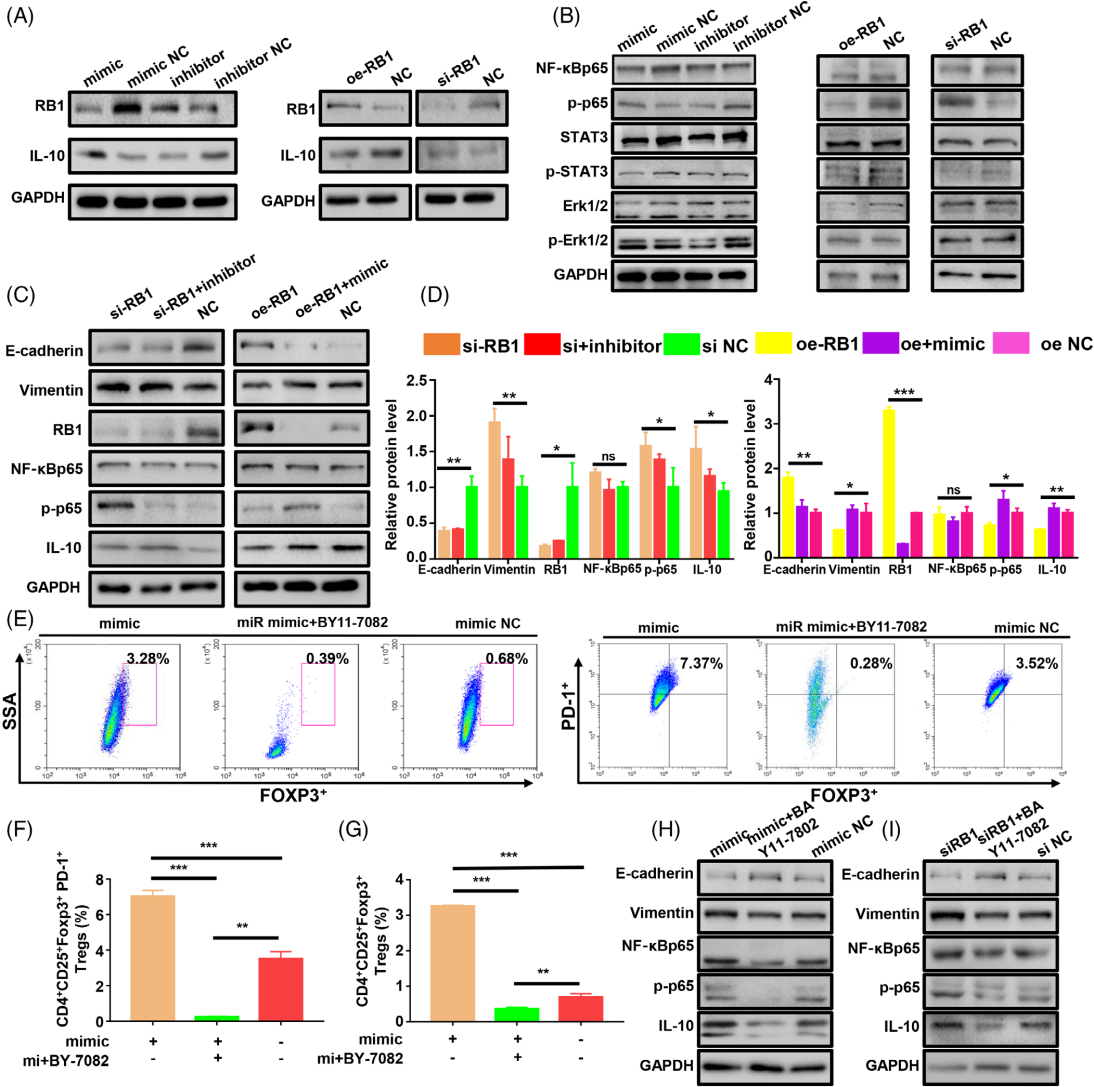
MiR‐192‐5p/RB1 contributes to the expression of IL‐10 by regulating NF‐κBp65. (A) The expression of RB1 and IL‐10 in the BGC‐823 cells or MKN45 cells transfected with miR‐192‐5p mimic/mimic NC/inhibitor/inhibitor NC/si‐RB1/oe‐RB1 were determined by western blot. (B) Western blot analysis of ERK1/2, p‐ERK1/2, STAT3, p‐STAT3, NF‐κBp65 and p‐NF‐κBp65 in the transfected BGC‐823 cells or MKN45 cells. (C) The expression of E‐cadherin, Vimentin, RB1, NF‐κBp65, p‐p65 and IL‐10 in the transfected BGC‐823 cells or MKN45 cells were analyzed by western blot. (D) After being co‐cultured with transfected GC cells or NF‐κBp65 inhibitor BAY11‐7082, the infiltration of FOXP3^+^Tregs was detected by flow cytometric. (E) The infiltration of PD‐1^+^Tregs was detected by flow cytometric after being co‐cultured with transfected GC cells or NF‐κBp65 inhibitor. (F) Quantification of FOXP3^+^ expression on Tregs. (G) Quantification of PD‐1^+^ expression on Tregs. Standard deviation (SD) (H) BGC‐823 cells were transfected with miR‐192‐5p mimic, mimic NC and NF‐κBp65 inhibitor; the expressions of NF‐κBp65, p‐p65 and IL‐10 were measured by Western blot. (I) Western blot of NF‐κBp65, p‐p65 and IL‐10 in the treated BGC‐823 cells. Data are pooled from three independent experiments. Statistical analysis between two groups was conducted using two‐tailed *t*‐test. One‐way ANOVA statistical tests were adopted for more than two groups. Error bars, standard deviation (SD). **p < .01, ***p < .001

Next, we explored the mechanisms via which miR‐192‐5p/RB1 regulates IL‐10 secretion. We searched for NF‐κB, ERK1/2, STAT3 pathways, which have been proven to be involved in regulating IL‐10 expression. Western blot (WB) results revealed that miR‐192‐5p and RB1 markedly affect phosphorylation levels of NNF‐κBp65 (p‐NF‐κBp65). While there was no distinct change in the phosphorylation expression of ERK1/2, STAT3 in miR‐192‐5p or RB1 treated cells. We then explore whether miR‐192‐5p/RB1 mediates its effects via the NF‐κBp65 pathway. WB assay was conducted to explore the expression of NF‐κBp65 phosphorylation (Figure 6C,D). The results showed that RB1 knockdown promoted NF‐κBp65 phosphorylation (*p* = .027), and the miR‐192‐5p inhibitor did not block this effect in GC cells (*p* = .185). Conversely, RB1 overexpression reduced NF‐κBp65 phosphorylation (*p* = .023), and this effect was counteracted by miR‐192‐5p (*p* = .01).

To examine whether IL‐10 is regulated by the NF‐κBp65, BAY11‐7082 (an inhibitor of NF‐κBp65, 10 μM) was used in the in vitro co‐cultivation. WB results suggested that blocking NF‐κBp65 with BAY11‐7082 neutralized IL‐10 induction by miR‐192‐5p overexpression (*p* < .001) or RB1 knockdown (*p* = .0009) (Figure 6H,I, Figure S12A,B). Flow cytometry results showed that BAY11‐7082 significantly reduced the ratio of the FOXP3^+^ (*p* < .001) and PD‐1^+^ Tregs (*p* < .001) in miR‐192‐5p overexpressing GC cells (Figure 6E–G). In addition, the proportions of FOXP3^+^ (*p* < .001) and PD‐1^+^ Tregs (*p* < .001) were also repressed by BAY11‐7082 in RB1 knockdown GC cells (Figure S8C,D). Collectively, the above results substantiated that the miR‐192‐5p/RB1 axis regulates IL‐10 secretion through the NF‐κBp65 signaling pathway in GC.

### 2.7 MiR192‐5p/RB1 suppresses the transcriptional activity of NF‐κBp65 and NF‐κBp65 in turn promotes miR‐192‐5p expression

**FIGURE 7:**
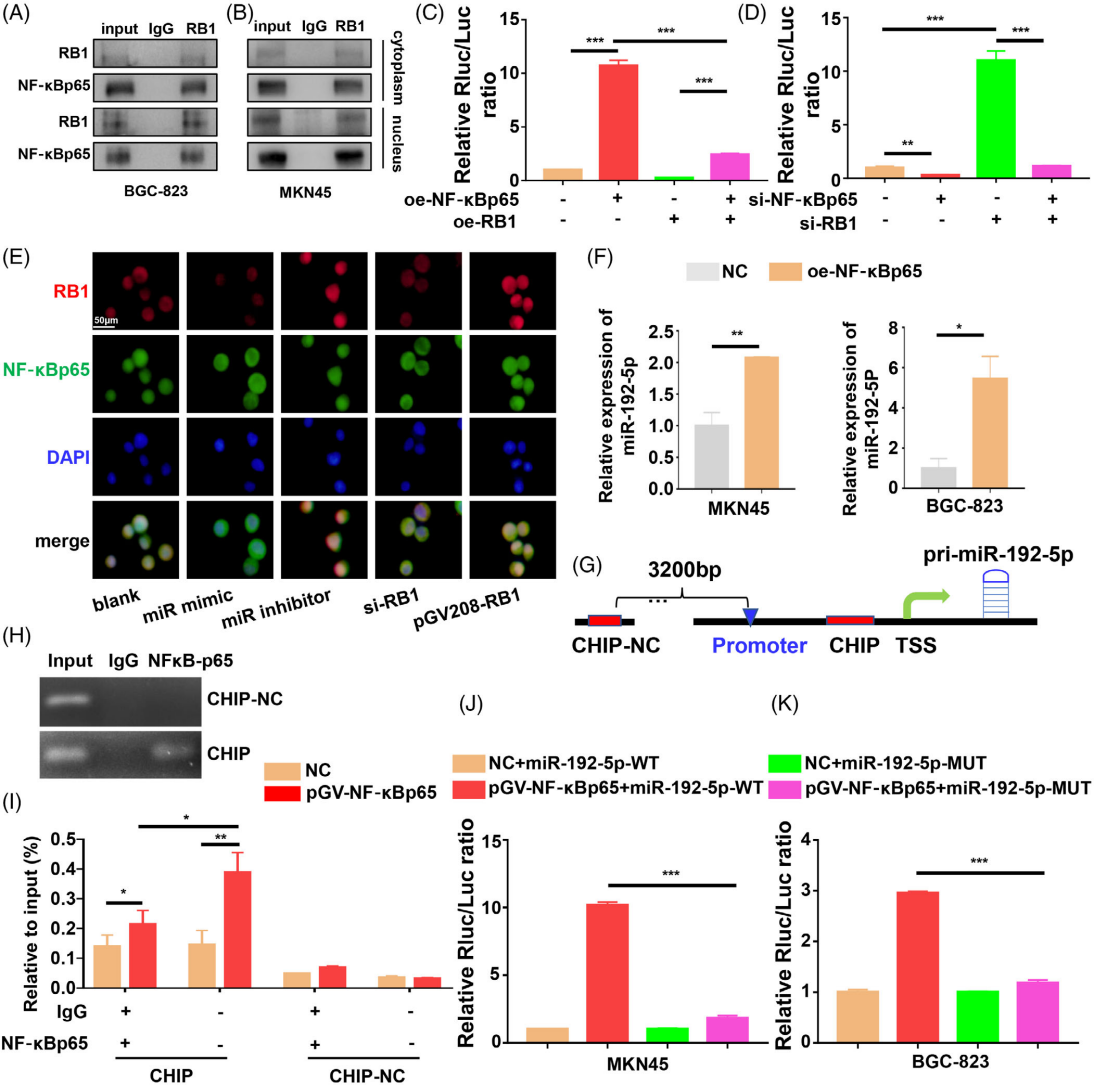
RB1 bound to NF‐κBp65 to inhibit its transcriptional activity. (A and B) Nuclear‐cytoplasmic fractionation and CoIP assay detected the interaction between RB1 and NF‐κBp65. (C and D) Luciferase assay of BGC‐823 or MKN45 cells transfected with luciferase reporter containing IL‐10 promoter. (E) The expression of RB1 and NF‐κBp65 were determined by immunofluorescence in gastric cancer (GC) cells transfected with miR‐192‐5p/RB1. (F) The miR‐192‐5p expressions of NF‐κBp65 overexpressed MKN45 cells and BGC‐823 cells were measured by qRT‐PCR. U6 was used for qRT‐PCR normalization of miR‐192‐5p. (G) Schematic illustration of the NF‐κBp65‐binding of miR‐192‐5p promoter. (H) Chromatin immunoprecipitation (ChIP) assay showed the direct binding of NF‐κBp65 to the miR‐192‐5p promoter, including non‐specific control (NC) and ChIP in GC cells. IgG ChIP was used as negative control. qRT‐PCR was conducted with the resulting precipitated DNA. Input, 2% of total lysate. (I) qRT‐PCR of the ChIP products confirmed the direct binding of NF‐κBp65 to the miR‐192‐5p promoter in GC cells. Input, 2% of total lysate. (J and K) Luciferase assay of BGC‐823 cells and MKN45 cells transfected with luciferase reporter containing miR‐192‐5p promoter. Data are pooled from three independent experiments. Statistical analysis between two groups was conducted using two‐tailed *t*‐test. One‐way ANOVA statistical tests were adopted for more than two groups. Error bars, standard deviation (SD). *p < .05, **p < .01, ***p < .001

RB1 played critical roles as transcriptional repressor and was reported to interact with the NF‐κBp65.^35^ Since we observed that miR‐192‐5p/RB1 could regulate IL‐10 production through the NF‐κBp65, we further investigated the underlying mechanism by which RB1 regulates NF‐κBp65. Firstly, nuclear‐cytoplasmic fractionation and co‐immunoprecipitation (CoIP) assay showed an endogenous interaction between RB1 and NF‐κBp65 (Figure 7A,B). The RB1 expression was higher in the nucleus than that in the cytoplasm and the RB1‐NF‐κBp65 binding primarily occurred in the nucleus. The IF results also showed that RB1 was present in the nucleus (Figure 7E). Thus, we speculated that RB1 binds to NF‐κBp65 to regulate the transcription of the NF‐κBp65 target gene in the nucleus. Luciferase activity was detected to confirm our observation (Figure 7C,D). The results revealed NF‐κBp65 activated the transcription activity that was driven by the IL‐10 promoter (*p* < .001), whereas RB1 dramatically suppressed it (*p* < .001). RB1 depletion increased the firefly luciferase expression (*p* < .001). Furthermore, after overexpression of NF‐κBp65, RB1 abolished the transcription activity of luciferase (*p* < .001). However, after knockdown of NF‐κBp65, RB1 depletion has little effect on the luciferase expression. In addition, miR‐192‐5p increased the firefly luciferase activity in GC cells with overexpressed RB1 (*p* < .001). After knockdown of RB1, miR‐192‐5p inhibitor has little impact on the luciferase activity (*p* = .061) (Figure S9A,B). These results suggest that RB1 binds to NF‐κBp65 in the nucleus to restrain the transcription of IL‐10.

Then we characterized the up‐regulation mechanism of miR‐192‐5p; PCR results revealed that miR‐192‐5p was highly expressed in NF‐κBp65 transfected BGC‐823 (*p* = .02) and MKN45 cells (*p* = .0069) (Figure 7F). To investigate whether miR‐192‐5p transcription was directly regulated by NF‐κBp65, we performed a chromatin immunoprecipitation (ChIP) assay (Figure 7G,H). The ChIP results showed great enrichment of NF‐κBp65 on the miR‐192‐5p promoter (*p* = .0129), which indicated that NF‐κBp65 binds to the miR‐192‐5p promoter. In addition, NF‐κBp65 increased the luciferase activity of the vector containing the miR‐192‐5p‐WT‐3′‐UTR, which was abrogated by miR‐192‐5p‐MUT‐3′‐UTR (*p* < .001) (Figure 7I,J). Overall, miR‐192‐5p/RB1 regulates NF‐κBp65 transcriptional activity, and NF‐κBp65 in turn promotes miR‐192‐5p expression, thus establishing a positive feedback loop.

### 2.8 MiR‐192‐5p/RB1 promotes tumour progression and immunosuppression in vivo

**FIGURE 8:**
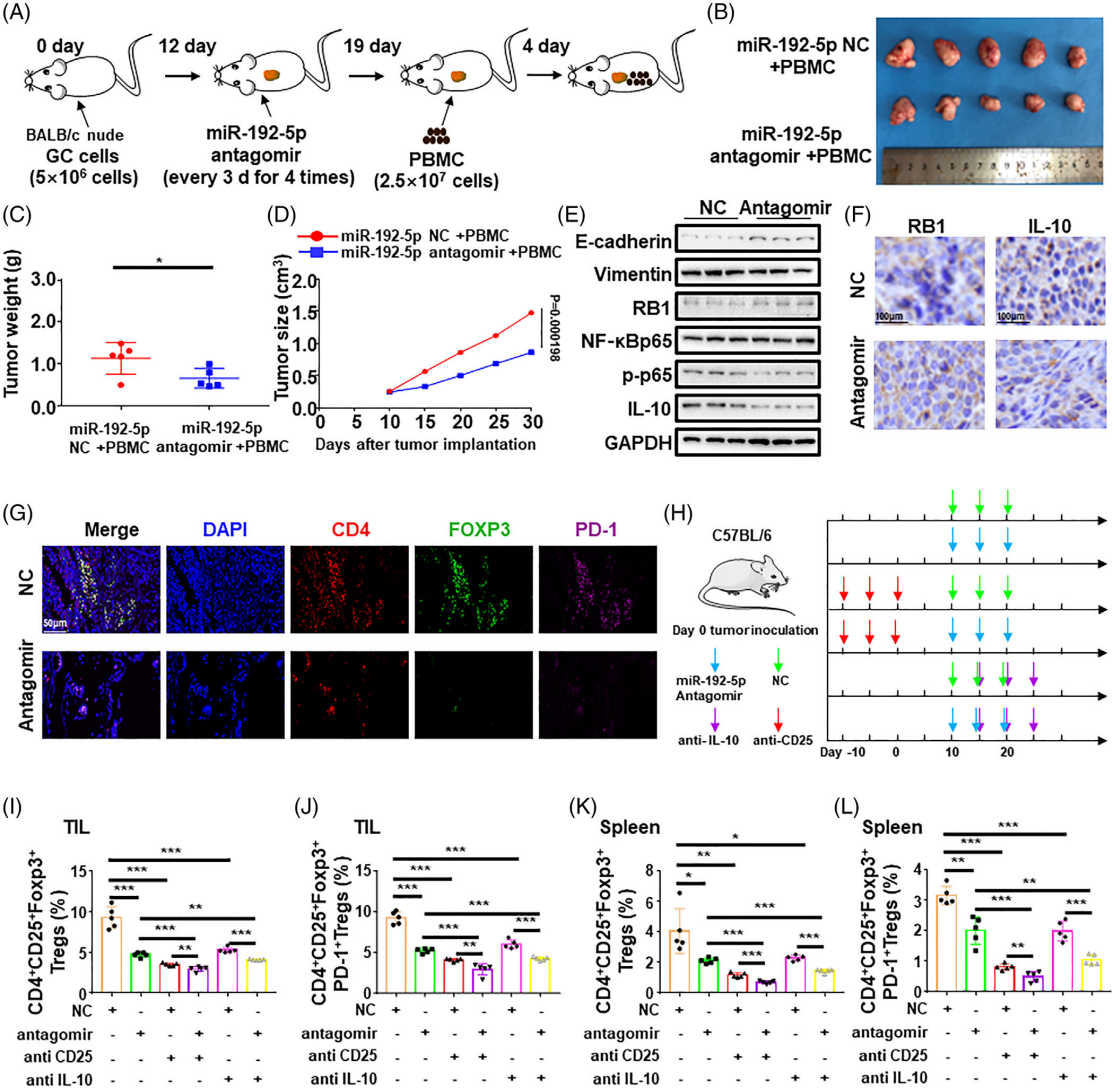
MiR‐192‐5p/RB1 contributes to tumour growth and FOXP3+Tregs infiltration in vivo. (A) A total of 5 × 10^6^ BGC‐823 cells were injected into the right flank of female BALB/c athymic nude mice. Twelve days after BGC cells injection, mice were injected intratumourally with miR‐192‐5p antagomir or antagomir negative control (NC). Seven days after miR‐192‐5p antagomir or antagomir NC injection, 2.5 × 10^7^ peripheral blood mononuclear cell (PBMC) cells were injected into tumour. Five weeks after BGC‐823 cells injection, mice were sacrificed. (B) The morphological characteristics of tumour xenograft, tumour size and tumour weight in miR‐192‐5p NC + PBMC or miR‐192‐5p antagomir + PBMC groups. (C) Tumour growth weight for two groups were measured; *n* = 5/group. (D) Analysis of tumour volume shows that miR‐192‐5p promotes xenograft tumour growth in nude mice. (E) Western blotting analysis of E‐cadherin, Vimentin, RB1, NF‐κBp65, p‐p65 and IL‐10 in xenograft tumours. Experiments were performed in triplicates. (F) Representative images of immunohistochemistry (IHC) staining for RB1 and IL‐10 expression in the xenograft tumours. Scale bar, 50 μm. (G) Immunofluorescence images of CD4 FOXP3 PD1 in the xenograft tumours. Scale bar, 50 μm. (H) Experimental overview of C57BL/6 tumour‐bearing mice. 5 × 10^5^ MFC cells were subcutaneously injected to the right flanks of C57BL/6 mice. Mice were randomly divided into six groups: miR‐192‐5p antagomir NC, miR‐192‐5p antagomir, anti‐CD25+miR‐192‐5p antagomir NC, anti‐CD25+miR‐192‐5p antagomir, anti‐IL‐10 + miR‐192‐5p antagomir NC and anti‐IL‐10 + miR‐192‐5p antagomir. Tumour growth was measured every 5d using a caliper and calculated by the formula (width^2^ × length)/2. MiR‐192‐5p antagomir/NC was injected into the tumour at a dose of 5 nmol/50 μl phosphate‐buffered saline (PBS) on day 10, 15, 20. Anti‐ CD25 mAb was injected i.p. 200 μg each mouse on day −10, −5, 0. Anti‐IL‐10 was administered i.p. 250 μg each mouse on day 15, 20, 25. *n* = 5/ group. (I) Evaluating FOXP3^+^ Tregs infiltration into the tumour. CD4, CD25 and FOXP3 were analyzed by flow cytometry. TILs, tumour infiltrating lymphocytes. *n* = 5/group. (J) Evaluating PD‐1^+^FOXP3^+^ Tregs infiltration into the tumour. CD4, CD25, PD‐1 and FOXP3 were analyzed by flow cytometry. *n* = 5/group. (K) Detecting FOXP3^+^ Tregs infiltration status of the spleen. *n* = 5/group. (L) Detecting PD‐1^+^FOXP3^+^ Tregs infiltration of the spleen. *n* = 5/group. Statistical analysis between two groups was conducted using two‐tailed *t*‐test. One‐way ANOVA statistical tests were adopted for more than two groups. Error bars, standard deviation (SD).*p < .05, **p < .01, ***p < .001

**FIGURE 9:**
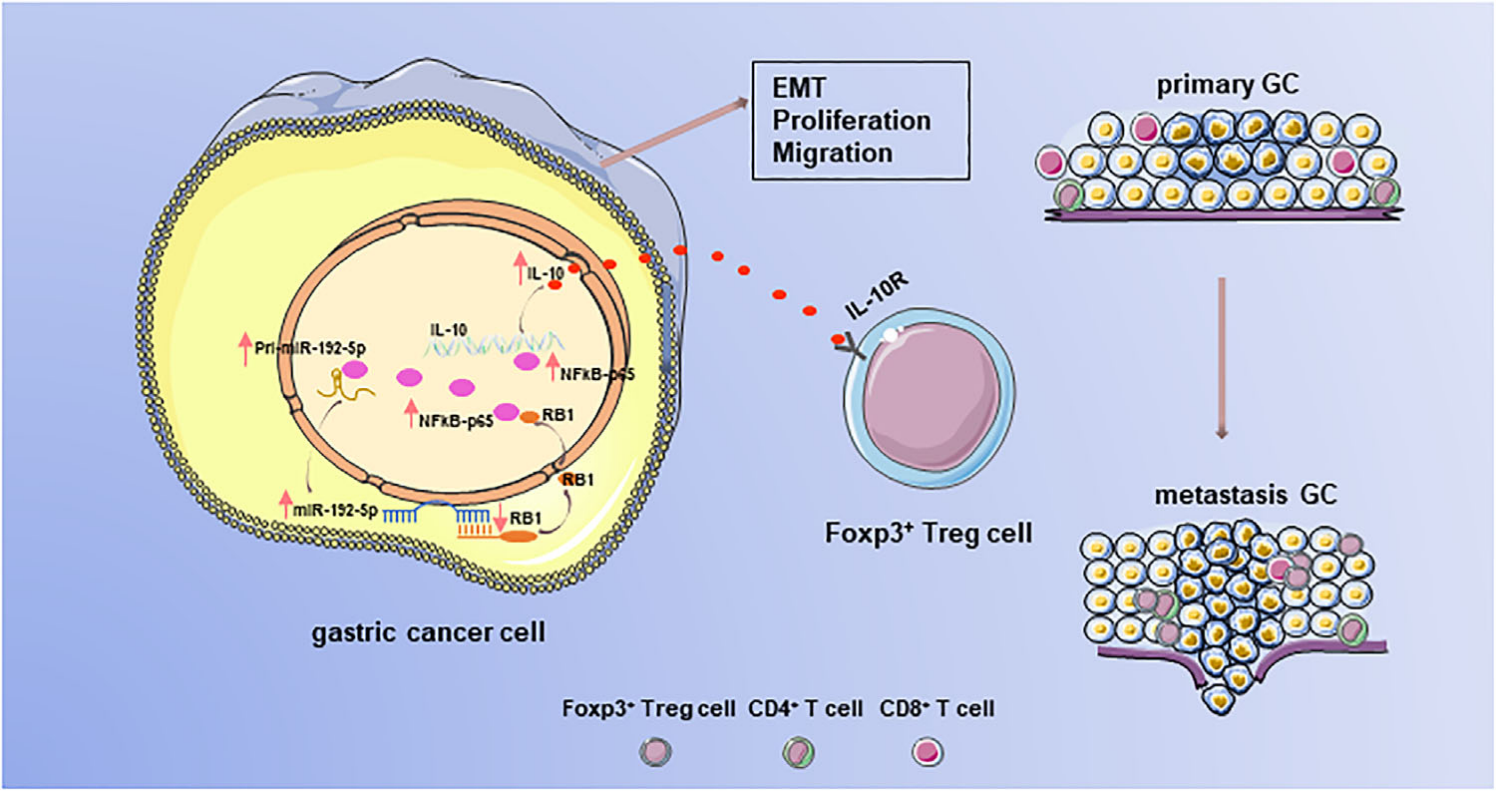
Graphical illustration of epithelial‐mesenchymal transition (EMT) gastric cancer (GC) cells acting on Tregs. MiR‐192‐5p/RB1/ NF‐κBp65 promoted tumour EMT and induced Treg cell differentiation by regulating IL‐10 secretion in the tumour microenvironment (TME), and NF‐κBp65 in turn promotes miR‐192‐5p expression in GC

To investigate the effects of miR‐192‐5p/RB1 on tumourigenesis and immunosuppression in vivo, we performed a subcutaneous tumour xenograft experiment using BGC‐823 cells. Nude mice were intratumourally injected with the miR192‐5p antagomir or miR192‐5p antagomir NC, respectively. After 7 days of miR192‐5p antagomir/NC injection, human PBMCs containing T cells were implanted into the tumour (Figure 8A). Consistent with the result in vitro, miR‐192‐5p inhibitor markedly suppressed tumour progression (*p* = .0413) (Figure 8B–D). The IL‐10 expression levels were declined in the miR‐192‐5p antagomir tumours (Figure 8E, Figure S10A,C). Additionally, miR192‐5p antagomir tumours had significantly elevated E‐cadherin and decreased vimentin compared to antagomir NC (Figure 8E, Figure S10A,C). Moreover, the miR192‐5p antagomir repressed the FOXP3 mRNA expression in tumour tissues (Figure S10A). IF results showed reduced accumulation of FOXP3^+^ and PD‐1^+^ FOXP3^+^Tregs in the tumours of the miR192‐5p antagomir group (Figure 8G, Figure S2B) (See Figure 9).

The effects of miR‐192‐5p/RB1 on Tregs were further explored with an established tumour model. FOXP3^+^ Tregs were depleted by anti‐CD25 antibody on day −10, −5 and 0. Then 5 × 10^5^ MFC tumour cells were implanted in C57BL/6 mice; mice were intratumourally injected with the miR‐192‐5p antagomir, antagomir NC, anti‐IL‐10 (Figure 8H). Figure S11A showed that tumour growth was significantly inhibited by miR‐192‐5p antagomir. Besides, Tregs depletion significantly reduced tumour burden. Mice in both antagomir and antagomir NC group treated with anti‐IL10 antibody showed protection against tumour growth. Analysis of tumour‐infiltrating lymphocytes revealed a significant fraction of FOXP3^+^ and PD‐1^+^FOXP3^+^Tregs in antagomir NC group compared with antagomir group (*p* < .001). Mice injected with the anti‐IL10 antibody showed declined the ratio of the FOXP3^+^ (*p* < .001) and PD‐1^+^ FOXP3^+^Tregs (*p* = .0001) in miR‐192‐5p antagomir and antagomir NC group (Figure 8I,J, Figure S11B). In addition, similar trend was observed for splenocytes (Figure 8K,L, Figure S11C). Therefore, these results corroborated that that miR‐192‐5p/RB1/NF‐κBp65/IL‐10 induces tumour EMT to enhance tumour growth and immunosuppression. In summary, our findings established that the miR‐192‐5p‐RB1‐NF‐κBp65 signaling pathway promotes tumour EMT, metastasis and Treg cell differentiation in GC (Figure 9).

## 3 DISCUSSION

The TME remains a major obstacle that impedes productive anti‐tumour immune responses. In this study, we observed that miR‐192‐5p affects tumour EMT by regulating RB1, inducing the secretion of IL‐10 to promote FOXP3^+^Treg cell differentiation and PD‐1 expression on Tregs in the TME. Moreover, miR‐192‐5p/RB1 induced PDL1 expression on GC cells, which could be a major contributor to the potent immunosuppressive GC microenvironment. Our research demonstrates the function and clinical significance of the miR‐192‐5p/RB1/NF‐κBp65 feedback loop in GC progression and immune escape.

MiRNAs are involved in cancer progression.^20^ MiR‐192‐5p expression varies across various cancers as oncogene or tumour suppressor gene.^26^, ^36^ Previous studies confirmed that miR‐192‐5p enhances tumour proliferation in prostate, esophageal and ovarian cancers.^37^, ^38^, ^39^ While there is recent evidence that miR‐192‐5p inhibits tumour metastasis in lung cancer.^40^ Such a contradiction might be due to the differences in tissues. In GC, miR‐192‐5p facilitates cell proliferation by regulating the Wnt signaling pathway.^41^ In our search, we confirmed that miR‐192‐5p promotes GC EMT, migration and invasion by targeting RB1. When miR‐192‐5p was depressed, these effects caused by RB1 depletion were relieved.

RB1 is a chromatin‐associated tumour suppressor that can limit the transcription of cell cycle genes.^42^ RB1 depletion has been shown to induce cell cycle defects, compromise G1/S cell cycle arrest and reduce senescence, which makes cells sensitive to oncogenic proliferation.^29^ RB1 is significantly associated with metastasis of various cancer.^45^ RB1 expression is reduced in GC,^46^ which has also been confirmed in our research. Additionally, we revealed that the RB1 affected EMT, cell proliferation, migration and invasion. EMT is regulated by different pathways including the TGF‐β, Wnt, PI3K/Akt and NF‐κB signaling pathway,^47^, ^48^ which promote the expression of transcription factors to drive the occurrence of EMT. It was reported that RB1 depletion activates the NF‐κBp65 and nuclear translocation of NF‐κBp65.^35^ RB1 specifically suppresses NF‐κBp65 activity and inhibits the expression of NF‐κB target genes, including PD‐L1.^49^ Additionally, it has been reported that RB1 affects the PI3K/Akt pathway to promote nasopharyngeal carcinoma EMT.^29^ RB1 can interacts with E2F1 to regulate EMT related proteins in lung cancer.^50^ Our study showed that knock down of NF‐κBp65 lessened the miR‐192‐5p/RB1‐mediated GC cell EMT, in addition, RB1 bound to NF‐κBp65 and inhibited its transcriptional activity. Thus, miR‐192‐5p/RB1‐mediated EMT may be attributed to NF‐κBp65.

The TME plays a vital role in immune regulation. Through GSEA analysis, we found that miR‐192‐5p/RB1 was linked to the Th17 cell differentiation pathway. Despite playing opposing roles in immune regulation, Treg and Th17 cells share a common differentiation pathway. We found that miR‐192‐5p/RB1 is correlated with Tregs but not Th17 cells in tumour tissues. Tregs can be classified into two types: natural/thymic‐derived (nTreg) and peripherally induced cell (pTreg).^51^ FOXP3 is a key regulatory gene for Treg development; CD4^+^CD25^+^FOXP3^+^ Tregs, either derived from nTreg or induced from pTreg, can suppress immune responses in TME.^52^ A wealth of evidence suggests that FOXP3^+^ Tregs infiltrate GC. Some studies showed that tumour‐infiltrating Treg cells can promote GC development and metastasis.^54^, ^55^, ^56^, ^57^ Tumour‐infiltrating Tregs inhibit anti‐tumour immune response via several pathways, including the production of immunosuppressive cytokines, PD‐1 checkpoint inhibition and up‐regulation membrane protein PD‐1 and CTLA‐4,^58^, ^59^ ultimately facilitating tumour metastasis.^60^ Tregs promote cancer cell invasion by affecting the EMT status.^61^, ^62^ In addition, Tregs promote tumour angiogenesis in the TME through the secretion of VEGF‐A and IL‐10, thereby supporting tumour metastasis.^63^ In vivo experiments, the tumour volume was significantly smaller after eliminating Tregs with anti‐IL‐10 or anti‐CD25, which confirmed the effect of Tregs on tumour progression. Several studies have reported compelling evidence that tumour cells produce various cytokines and chemokines to promote the proliferation of Tregs and induce FOXP3^–^ T cells into FOXP3^+^ Tregs.^64^, ^65^ It was well established that the Treg cell differentiation is induced by the TGF‐β and IL‐10 cytokines.^66^ Therefore, the expression levels of these cytokines in the supernatants of GC cells co‐cultured with Tregs were detected. IL‐10 was significantly increased in the supernatants of the co‐culture system. NF‐κBp65 binds to upstream of the IL‐10 and has a role in enhancing IL‐10 expression.^68^ We found that there is an RB1/NF‐κBp65/IL‐10 axis in GC cells that induces Treg cell differentiation. EMT tumour cells have a close interaction with Tregs, resulting in further tumour growth and metastatic spread.^16^ Our study provides evidence for the vital role of IL‐10 induced by EMT tumour cells in the creation of a favourable TME for GC progression through regulating Treg cell differentiation.

Our data showed that miR‐192‐5p/RB1 can activate the PD‐1/PDL1 pathway. Zhang et al. revealed that PD‐1 is indispensable for Tregs suppressive functions, and loss of PD‐1 expression impaired the function of Tregs.^69^ Nair et al. found that PD‐1 enhances the stability of Tregs by increasing FOXP3 expression.^70^ Cancer cells can evade immune surveillance by expressing co‐inhibitory molecule PDL1; Tregs express inhibitory molecule such as PD‐1, which interacted with co‐inhibitory molecule PDL1, resulting in attenuated CD8^+^ T cell responses.^71^ We found that miR‐192‐5p/RB1 promoted the PDL1 expression on tumour cells and increased the percentage of PD‐1^+^FOXP3^+^ Tregs, which may enhance the immunosuppressive capacity of Tregs and promote immune evasion in GC.

Although some studies have confirmed that Tregs promoted GC metastasis, there is a primary limitation of our study that an absence of corroboration that Tregs deficiency inhibits tumour progression by affecting immune cells and tumour cells. Therefore, additional work is needed to understand its underlying mechanism. Additionally, since we found that miR‐192‐5p/RB1 could activate the PD‐1/PDL1 pathway, further studies are needed to explore the mechanism by which miR‐192‐5p/RB1 regulates PD‐1/PDL1 pathway and might uncover additional therapeutic target in GC.

In summary, we revealed that miR‐192‐5p promotes GC EMT, thereby promoting malignancy and FOXP3^+^ Treg cell differentiation via RB1/NF‐κBp65/IL‐10 axis, thereby highlighting the miR‐192‐5p/RB1/NF‐κBp65/IL‐10 pathway as a new therapeutic strategy for tumour immunotherapy in GC.

## 4 MATERIALS AND METHODS

Detailed materials and methods are provided in Supplemental Materials.

### 4.1 Patient samples

Thirty paired GC samples were obtained from patients diagnosed with GC based on pathological examinations. GC patients had received tumour radical surgical treatment at the Zhongnan Hospital, which is affiliated with Wuhan University. No patient had received radiotherapy or chemotherapy before surgery. Peripheral Blood (PB) samples (3 ml) were collected in tube containing EDTA from 30 GC patients and 40 healthy volunteers without any malignancy (Table S2). The study was approved by the Ethics Committee of Zhongnan Hospital, Wuhan University. All patients gave informed consent.

### 4.2 PBMC isolation and activation

PBMCs were obtained from voluntary donor by venipuncture. Blood (3 ml) was diluted with 3‐ml phosphate‐buffered saline (PBS), layered on equal volume of Lymphoprep solution (Dakewe, #711011), and centrifuged at 700 × g for 20 min. PBMCs were washed with 6‐ml PBS then resuspended in 1640 medium (with 10% heat‐inactivated Fetal Bovine Serum). Soluble anti‐CD3, anti‐CD28, human interleukin‐2 (IL‐2) (PeproTech, USA, #200‐02) added to cells at concentrations 2 μg/ml, 2.5 μg/ml and 200 ng/ml.

### 4.3 Flow cytometry

For PBMCs, FITC‐CD4, APC‐CD25 or APC‐CY7‐CD25, APC‐PD‐1 (Biolegend, USA) were added in 5 × 10^5^ PBMC and then incubated for 45 min at 4°C. Cells were centrifuged, fixed, permed according to the instructions of Fix&Perm kit (Biolegend, USA, # 421403). Then, PE‐Foxp3 (Biolegend, USA) was added for 45 min. Cells were detected on the flow cytometer.

For spleen and tumour, tumour was cut into 2‐mm sized pieces and digested in 1 mg/ml collagenase type IV (BioFroxx, #2091MG100, CAS 9001‐12‐1), incubated at 37°C for 1 h, passed through 70‐mm filter and suspended in PBS (.5–1 × 106 cells/ml). Spleen was filtered and suspended in PBS (.5–1 × 106 cells/ml). Then the cell suspension was stained for flow cytometry. The antibodies were all purchased from Biolegend and shown in Table S6.

### 4.4 Luciferase gene reporter assay

The promoter region of the RB1 gene containing miR‐192‐5p wild‐type or mutated‐type binding sequence was inserted into the pmiR‐RB‐ReportTM Vector. MKN45 and BGC823 cells were transfected with vectors or miR‐192‐5p mimic/NC by Lipofectamine 2000 (Invitrogen, #11668030). Luciferase Reporter Assay System (Promega, USA, #E1910) was used for detecting the luciferase activity. For detecting the luciferase activity of NF‐κBp65, the GC cells were transfected with vector containing the NF‐κBp65 binding site of IL‐10 promoter. To examine the binding site between NF‐κBp65 and miR‐192‐5p, tumour cells were transfected with vectors containing miR‐192‐5p binding site and NF‐κBp65.

### 4.5 RIP assay and ChIP assay

A RIP Kit (Millipore, MA, USA, #17‐700) was used for RIP assay. Briefly, 4 × 10^7^ cells were lysed then incubated with human antiAGO2 antibody at 4°C for overnight. Normal Mouse IgG (Millipore, MA, #17‐700, USA) was used as negative control. Co‐immunoprecipitated RNA was extracted for qRT‐PCR. A Chromatin IP Kit (Cell Signaling Technology, CST, #9002, USA) was used for ChIP assay. Anti‐NF‐kBp65 (CST, USA, #8242) and normal rabbit IgG (CST, USA, #3900) were used. Immunoprecipitated chromatin was measured by qRT‐PCR.

### 4.6 CoIP assay

IP/CoIP Kit (#abs955, Shanghai, China) was used to perform CoIP assay. GC cells were lysed on ice and centrifuged at 4°C for 10 min. Supernatants were collected and incubated with anti‐RB1 (CST, #9309, USA) or normal rabbit IgG (CST, #3900, USA) with rotating at 4°C overnight. On the following day, Protein A/G was added to the supernatants with rotation at 4°C for 3 h. Afterward, supernatants were subjected to WB analysis.

### 4.7 Subcutaneous tumourigenesis experiment

Animal experiment was conducted in line with the Guide for the Care and Use of Laboratory Animals of Wuhan University. BALB/c nude mice (female, 4‐week‐old) and C57BL/6 mice (female, 4‐week‐old) were purchased from Hubei Research Center of Laboratory Animals (Wuhan, China,). Note that 5 × 10^6^ BGC‐823 cells were injected subcutaneously on the right flank region of the nude mice. After 12 days, miR‐192‐5p antagomir/NC (RiboBio, #miR30000222‐4‐5, China) was injected into the tumour at a dose of 5 nmol/50 μl PBS every 3 d for four times. Then PBMCs obtained from healthy volunteers were injected into tumour at the dose of 2.5 × 10^7^ cells/50 μl after a week. Ten days after BGC cell injection, tumour volumes were measured with a caliper every 5 days and calculated by the formula (width^2^ × length)/2. The mice were euthanized after 5 weeks of BGC cell injection. The tumour tissues were collected for tumour weight and other analyses.

For C57BL/6 mice, on day −10, −5 and day 0, 10 mice were intraperitoneally (i.p.) injected with anti‐mouse CD25 monoclonal antibody (mAb). On day 0, 5 × 105 MFC cells were subcutaneously injected to the right flanks of mice. On day 10, mice were randomly divided into different groups: antagomir NC, miR‐192‐5p antagomir, anti‐CD25+ antagomir NC, anti‐CD25+miR‐192‐5p antagomir, anti‐IL‐10 + antagomir NC and anti‐IL‐10 + miR‐192‐5p antagomir. Tumour growth was measured every 5 days using a caliper and calculated by the formula (width^2^ × length)/2. On Day 30, mice were euthanized, spleens and tumours were collected for flow cytometry. MiR‐192‐5p antagomir/NC (RiboBio, # miR30000517‐4‐5, China) was injected into the tumour at a dose of 5 nmol/50 μl PBS on day 10, 15 and 20. Anti‐ CD25 mAb (BioXcell, #BE0012‐1MG, West Lebanon, NH, USA) was injected i.p. 200 μg each mouse on day −10, −5, and 0. Anti‐IL‐10 (Biolegend, clone JES5‐2A5, #504908, USA) was administered i.p. 250 μg each mouse on day 15, 20, 25.

### 4.8 Bioinformatics assay and statistics analysis

MiRNA expression was obtained from the GEO website and was analyzed by GEO2R. Heatmap was performed by Funrich v3.1.3. GSEA was conducted by Sangerbox (http://www.sangerbox.com/). The survival curve of RB1 was obtained from the Kaplan–Meier Plotter website (http://kmplot.com/analysis/). Eight hundred seventy‐five patients were included, and six patients were excluded in the Kaplan–Meier Plotter website.^72^ The association between miR‐192‐5p and clinical pathology parameters were analyzed by χ^2^ test or Fisher exact test. Univariable and multivariable Cox regression analyses were applied to detect potential risk factor. Spearman correlation was performed to study the correlation of two continuous variables. Kaplan‐–Meier analysis was used for evaluating survival curves. Data were shown as mean ± standard deviation. Statistical analyses were performed by SPSS 17.0 (SPSS Inc., USA). Statistical significance was determined by Student's *t*‐test. *p* < .05 was determined statistically significant.

## Supporting information

Supporting Information

## Data Availability

The data that support the findings of this study are openly available in Gene Expression Omnibus (GEO), reference number GSE78775, GSE86226, GSE164174. Other data that support our findings are available at the Clinical and Translational Medicin's website.
