# Supplementary material for: MiR‐192‐5p/RB1/NF‐κBp65 signaling axis promotes IL‐10 secretion during gastric cancer EMT to induce Treg cell differentiation in the tumour microenvironment
Source: Clin Transl Med. 2022 Aug 15;12(8):e992. doi: 10.1002/ctm2.992 (PMC9377151; doi:10.1002/ctm2.992)
Supplement: Supplementary file 1 — Supporting Information [file CTM2-12-e992-s001.docx]

Supplementary Materials for

**MiR-192-5p/RB1/NF-κBp65 signaling axis promotes IL-10 secretion during gastric cancer EMT to induce Treg cell differentiation in the tumor microenvironment**

Jialin Song^1,2,3†^, Zaihuan Lin^1,2,3†^, Qing Liu^4,5†^, Sihao Huang^1,2,3†^, Lei Han^1,2,3^, Yan Fang^6^, Panyi Zhong^1,2,3^, Rongzhang Dou^1,2,3^, Zhenxian Xiang^1,2,3^, Jinsen Zheng^1,2,3^, Xinyao Zhang^1,2,3^, Shuyi Wang^1,2,3 *^, and Bin Xiong^1,2,3 *^

+ These authors contributed equally to this work.

*** Correspondence**: Bin Xiong, Ph.D. E-mail: [binxiong1961@whu.edu.cn](mailto:binxiong1961@whu.edu.cn); Shuyi Wang, Ph.D. E-mail: [shuyiwang@whu.edu.cn](mailto:shuyiwang@whu.edu.cn).

**Financial support**: This work was supported by grants from the Health Commission of Hubei Province Scientific Research Project (No. WJ2019H012), Improvement Project for Theranostic ability on Difficulty miscellaneous disease (Tumor)(No. ZLYNXM202018), and National Natural Science Fund Youth Fund of China (No. 81702411)

**Conflict of interest disclosure statement**: The authors declare no potential conflicts of interest.


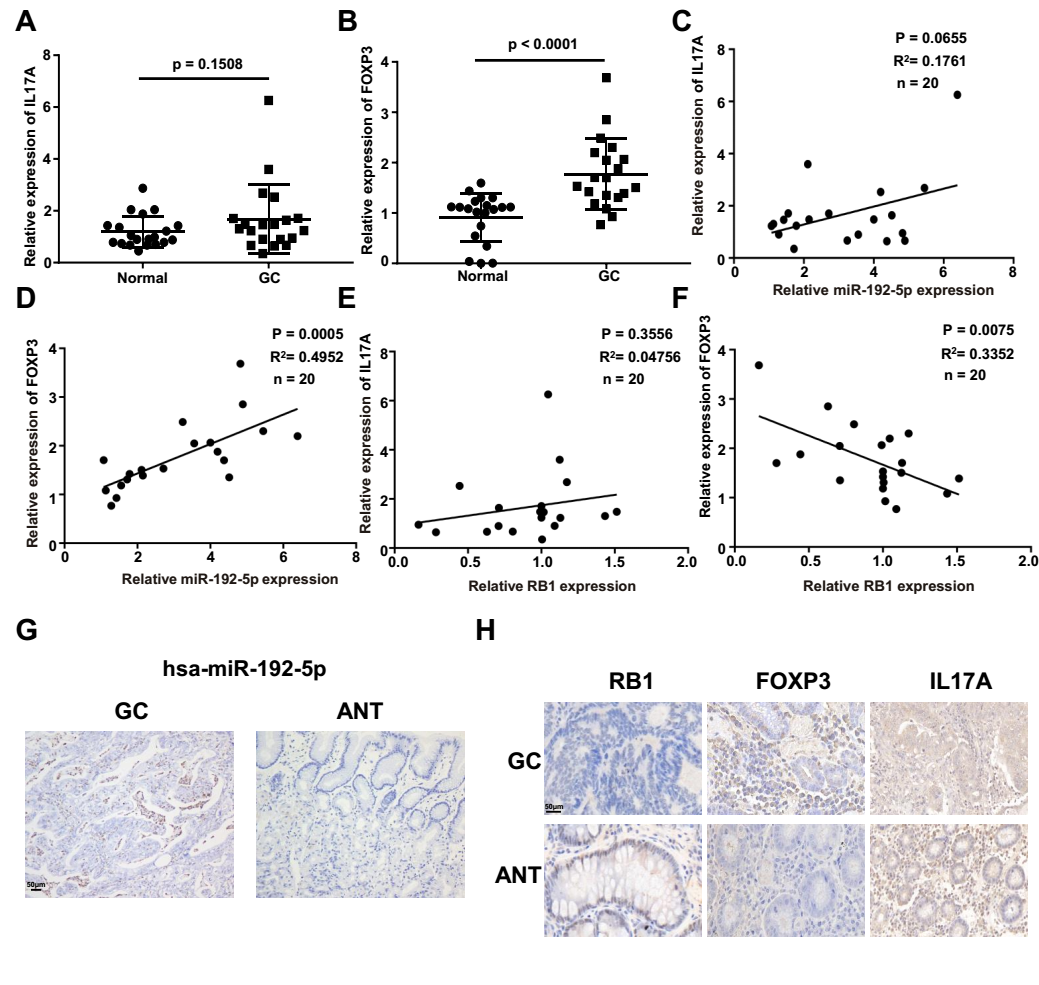


**Figure S1.** MiR-192-5p/RB1 was relevant to Tregs, but not Th17 cells in GC. **(A)** IL17A expression was not different between GC and ANT. GAPDH was used for qRT-PCR normalization. **(B)** qRT-PCR result showed that FOXP3 expression was considerably different between GC and ANT. GAPDH was used for qRT-PCR normalization. **(C)** Correlation analysis between IL17A and miR-192-5p expression of GC patients. **(D)** Correlation analysis between FOXP3 and miR-192-5p expression of GC patients. **(E)** Correlation analysis between IL17A and RB1 expression of GC patients. **(F)** Association of FOXP3 with RB1 expression of GC patients. **(G)** Representative CISH image of miR-192-5p in GC sample. Bar = 50μm. (H) IHC staining of RB1, FOXP3 and IL17A in GC sample. Bar = 50μm. Statistical analysis between two groups was conducted using two-tailed t-test. Error bars, SD.


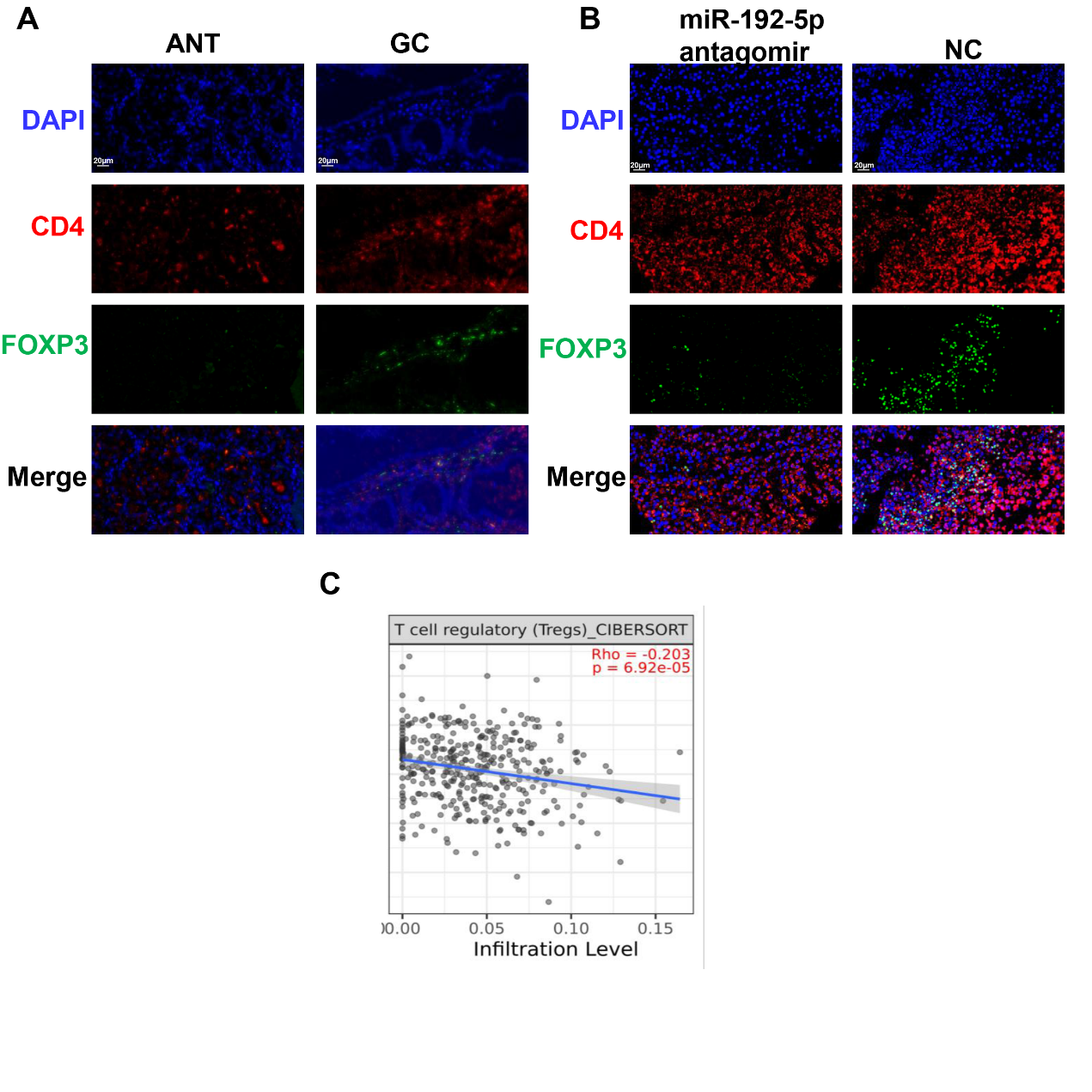


**Figure S2.** Increased number of infiltrating Treg cells in tumor tissues of GC. **(A)** Representative immunofluorescence photographs for Treg cells in GC and ANT. Bar = 20μm. **(B)** Representative immunofluorescence images for Treg cells in xenograft tumors. Bar = 20μm. **(C)** TIMER2.0 analysis of the correlation between RB1 and Treg cells infiltration in GC.


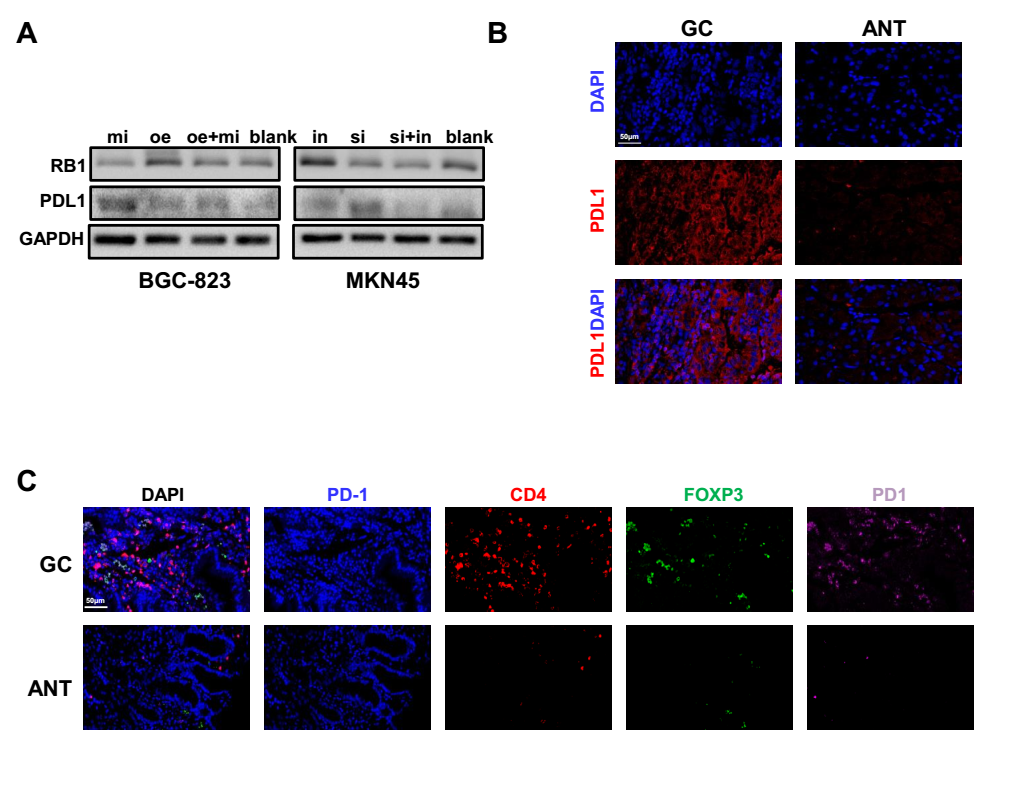


**Figure S3.** MiR-192-5p/RB1 increased PDL1 expression of GC cells. **(A)** Western Blot of PDL1 protein level in GC cells transfected with miR-192-5p/RB1. **(B)** Representative immunofluorescence photographs for PDL1 expression in GC and ANT. Bar = 50μm. **(C)** Representative immunofluorescence staining images for PD-1 in GC and ANT. Bar = 50μm. Data are pooled from three independent experiments.


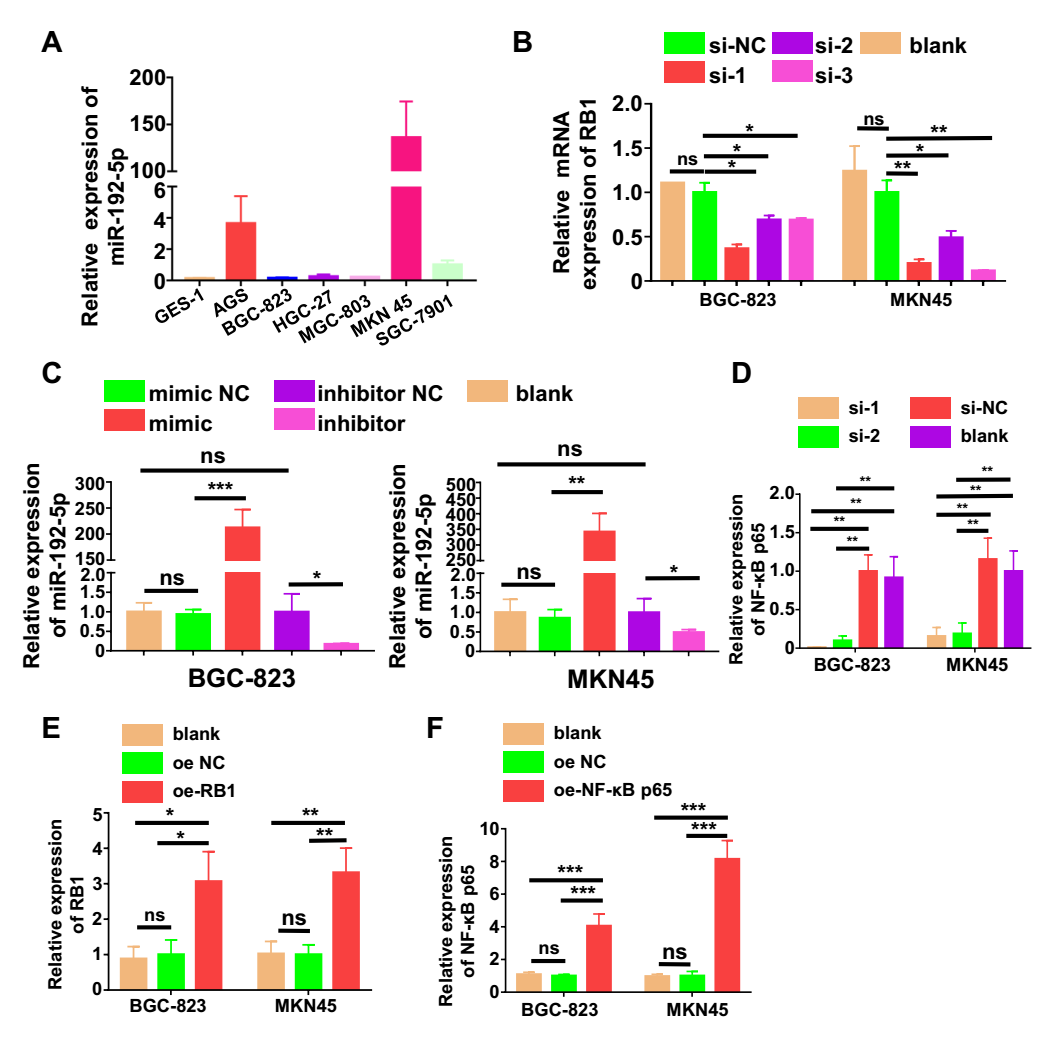


**Figure S4.** MiR-192-5p and RB1 expression in different cells. **(A)** MiR-192-5p expression in GC cells and GES-1 cells was detected by qRT-PCR. U6 was used for qRT-PCR normalization. **(B**)qRT-PCR analysis of the transfection efficacy of siRNA on RB1 expression in BGC and MKN45 cells. GAPDH was used for qRT-PCR normalization. **(C) q**RT-PCR analysis of the transfection efficacy of mimic and inhibitor on miR-192-5p expression in BGC and MKN45 cells. U6 was used for qRT-PCR normalization. **(D) q**RT-PCR analysis of the transfection efficacy of two siRNA on NF-κBp65 expression in BGC and MKN45 cells. GAPDH was used for qRT-PCR normalization. **(E) q**RT-PCR analysis of the transfection efficacy of RB1 overexpression in GC cells. GAPDH was used for qRT-PCR normalization.  **(F) q**RT-PCR analysis of the transfection efficacy of NF-κBp65 overexpression in GC cells. GAPDH was used for qRT-PCR normalization. Data are pooled from three independent experiments. Statistical analysis between two groups was conducted using two-tailed t-test. Error bars, SD. *P < 0.05, **P < 0.01, ***P < 0.001. ns: no significance.


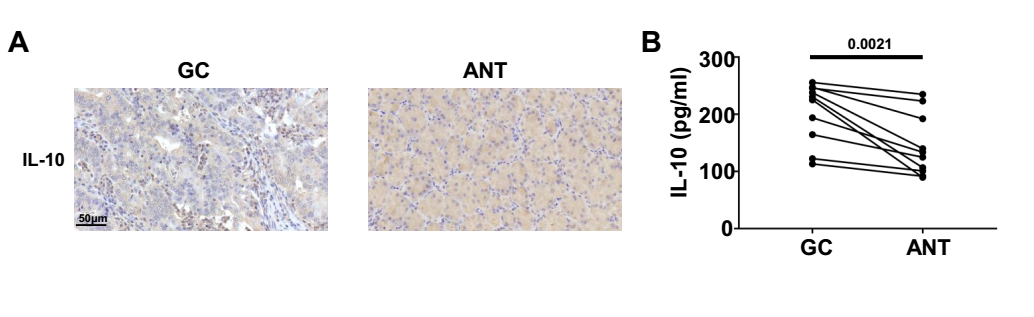


**Figure S5.** IL10 expression in GC and ANT. **(A)** IHC staining of IL10 expression in GC and ANT. Bar = 50μm. **(B)** ELISA analysis of IL10 secretion in 10 paired GC and ANT. Statistical analysis between two groups was conducted using two-tailed t-test.


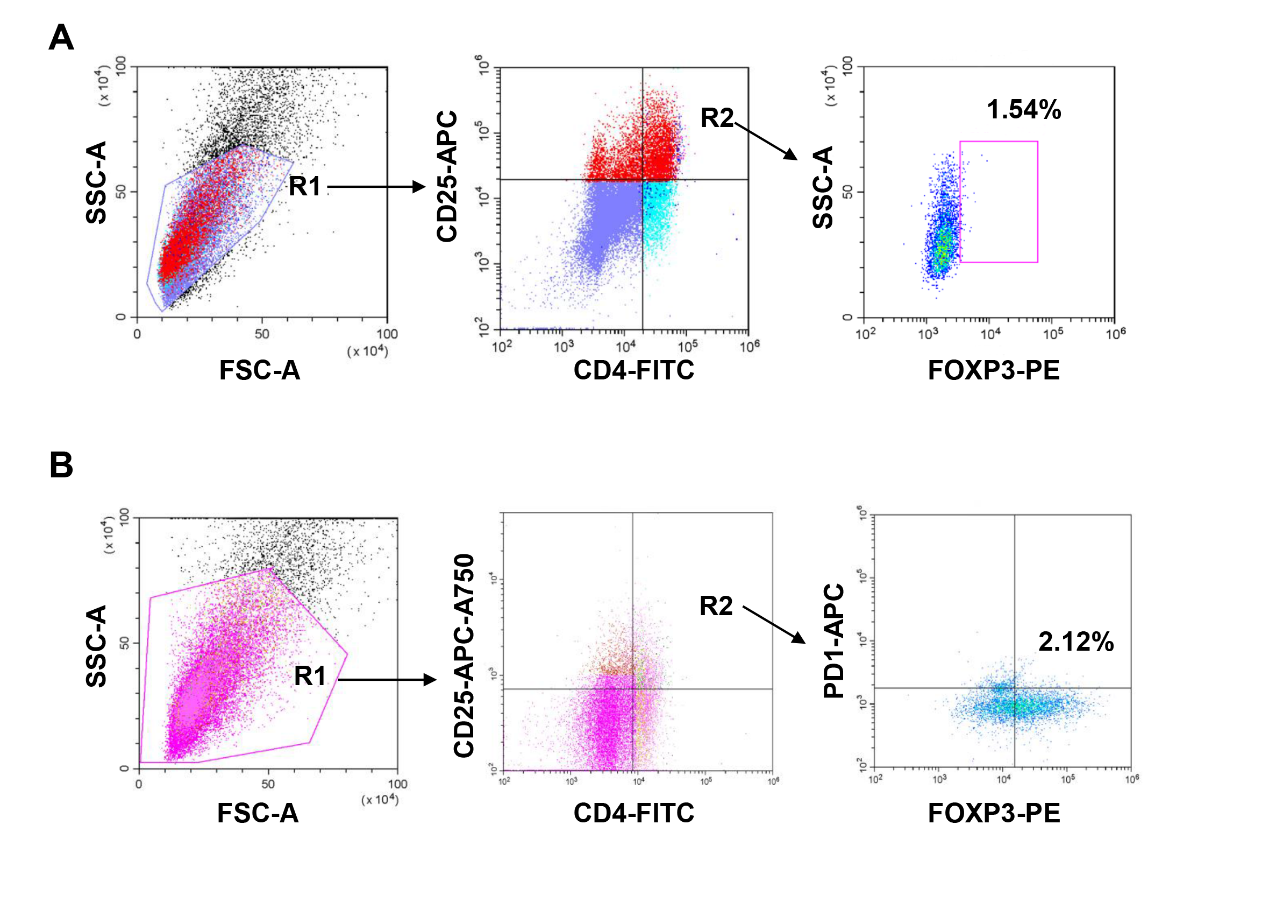


**Figure S6.** The gating strategy for Treg cell population. **(A)** To detect the proportion of FXOP3+ Tregs, the lymphocytes (R1) were gated according to their side- and forward-scatter properties, the CD4^+^CD25^+^ T cells (R2) were gated from R1, and CD4^+^CD25^+^FXOP3^+^ Tregs were gated from R2. **(B)** To detect the proportion of FXOP3+PD-1^+^ Tregs, the lymphocytes (R1) were gated according to the side-scatter and forward-scatter properties, the CD4^+^CD25^+^ T cells (R2) were gated from R1, and FXOP3^+^PD-1^+^ Tregs were gated from R2.


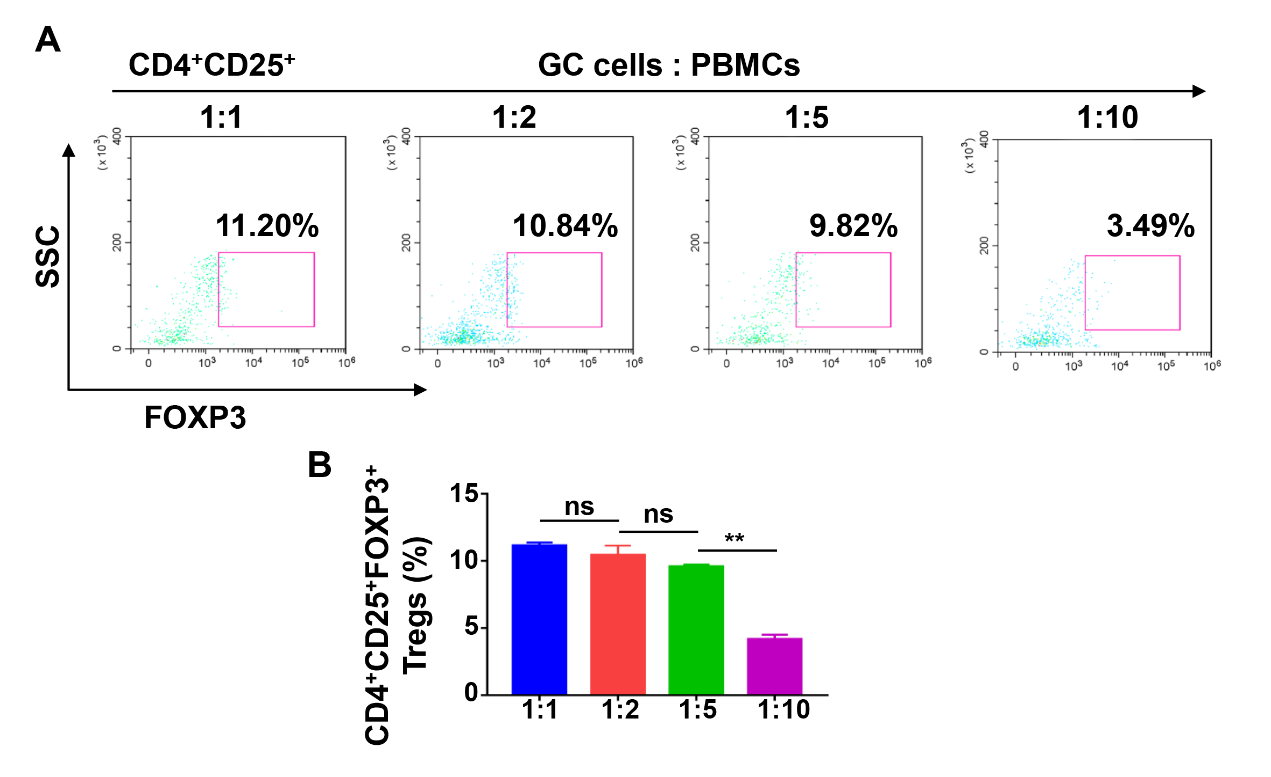


**Figure S7.** The co-culture system of GC cells and PBMCs. **(A)** The co-culture ratio of GC cells and PBMCs was 1:1,1:2,1:5, and 1:10, and the proportion of Tregs was detected by flow cytometry **(B)** Quantification of FOXP3+ Tregs. The proportion of Tregs in 1:1 group was the highest among all groups. Data are pooled from three independent experiments. Statistical analysis between two groups was conducted using two-tailed t-test. One‐way ANOVA statistical tests were adopted for more than two groups. Error bars, SD. ns: no significance. **P < 0.01.


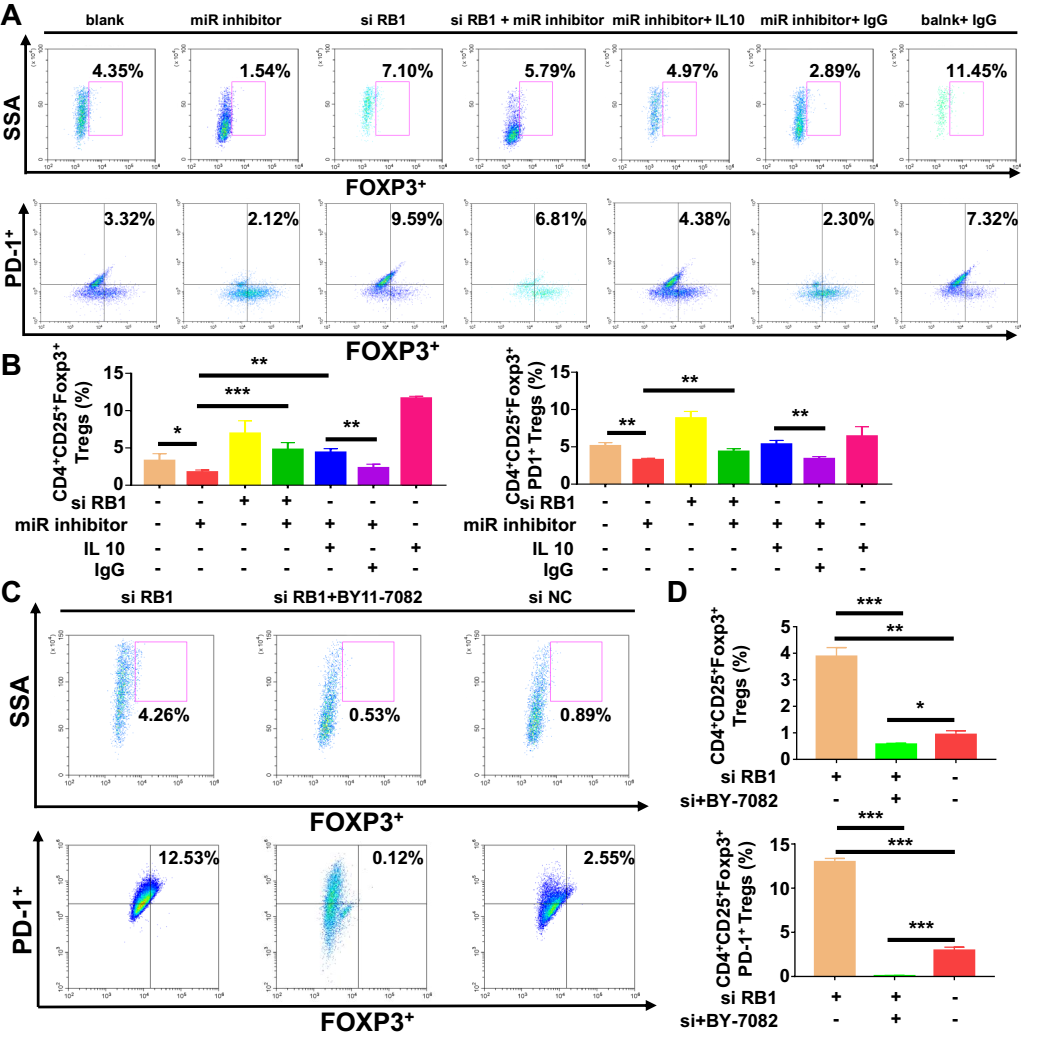


**Figure S8.** MiR-192-5p/RB1 contributes to the expression of IL10 by regulating NF-κBp65, thereby promoting Treg cell differentiation. **(A)** Flow cytometric of FOXP3+ Treg cells and PD-1+ Treg cells after co-cultured with transfected GC cells. **(B)** Quantification of FOXP3+ and PD-1+ expression of Treg cells. **(C)** After being co-cultured with transfected GC cells or BY11-7082 (10μM), the infiltration of FOXP3+Treg cells and PD-1+ Treg cells were detected by flow cytometric. **(D)** Quantification of FOXP3+ and PD-1+ expression on Treg cells. Data are pooled from three independent experiments. Statistical analysis between two groups was conducted using two-tailed t-test. One‐way ANOVA statistical tests were adopted for more than two groups. Error bars, SD. *P < 0.05, **P < 0.01, ***P < 0.001.


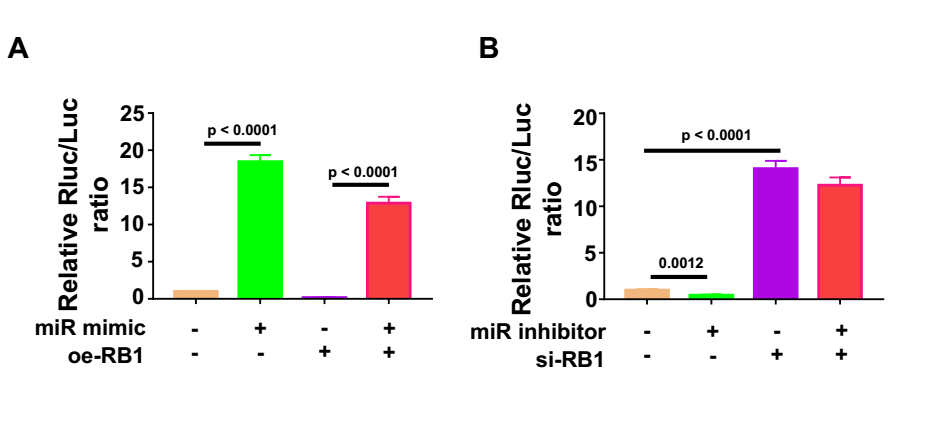


**Figure S9.** RB1 bound to NF-κBp65 to inhibit its transcriptional activity. **(A)** Luciferase assay of BGC-823 cells transfected with luciferase reporter containing IL10 promoter. **(B)** Luciferase assay of MKN45 cells transfected with luciferase reporter containing IL10 promoter. Data are pooled from three independent experiments. Statistical analysis between two groups was conducted using two-tailed t-test. One‐way ANOVA statistical tests were adopted for more than two groups. Error bars, SD.


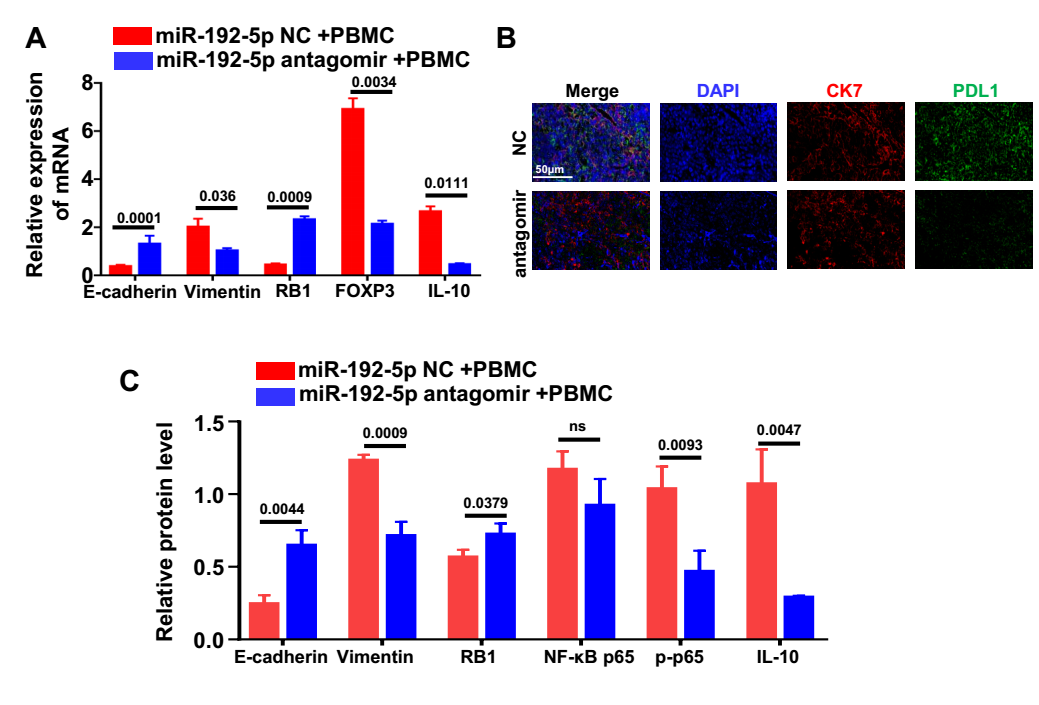


**Figure S10.** MiR-192-5p/RB1 contributes to EMT and PDL1 expression of tumors in vivo. **(A)** qRT-PCR analysis of E-cadherin, Vimentin, RB1, FOXP3 and IL-10 expression in xenograft tumors. GAPDH was used for qRT-PCR normalization of mRNA. **(B)** The expression of PDL1 was determined by Immunofluorescence in miR-192-5p antagomir group and NC group. **(C)** Experiments were performed in triplicates. Statistical analysis between two groups was conducted using two-tailed t-test. One‐way ANOVA statistical tests were adopted for more than two groups. Scale bar, 50μm. Error bars, SD. n = 5/group. ns, no significance.


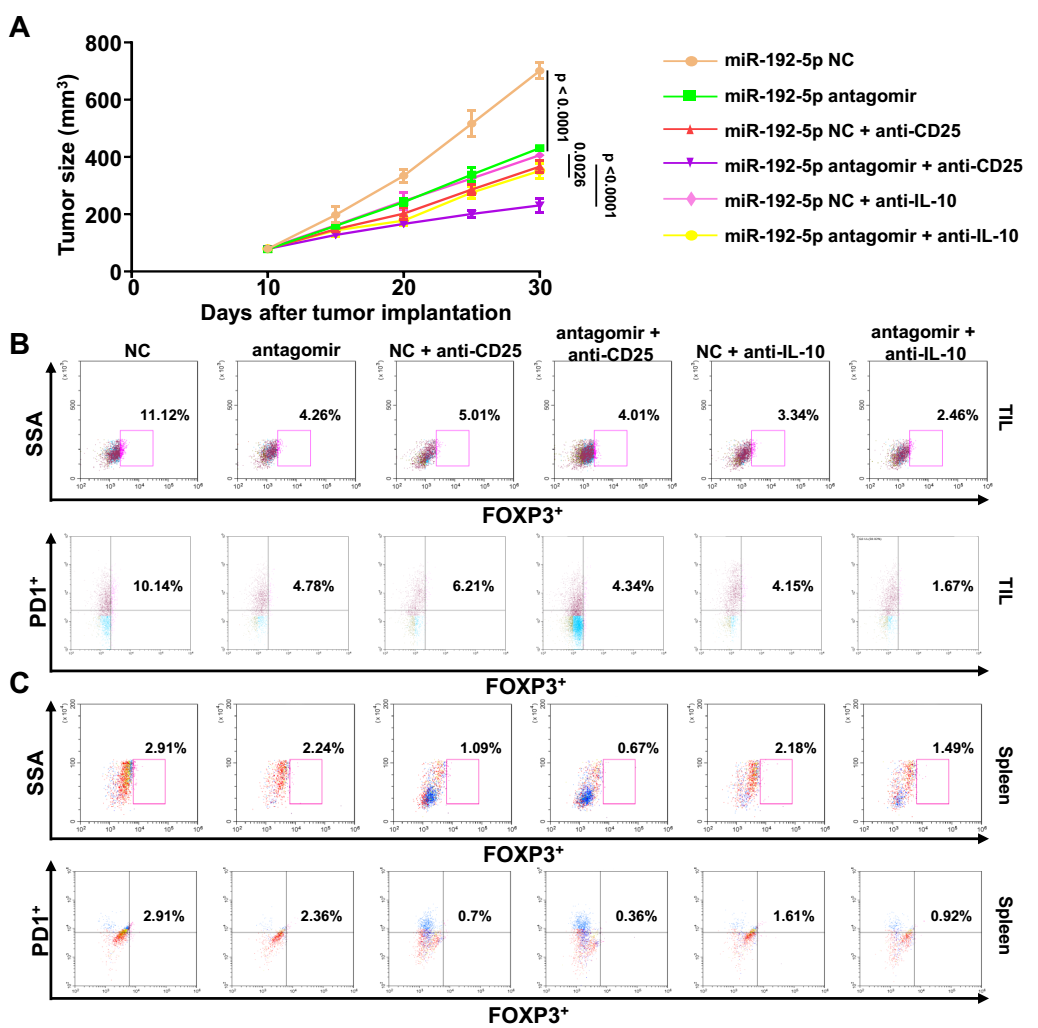


**Figure S11.** MiR-192-5p/RB1 promotes tumor growth and Tregs infiltration in vivo. **(A)** Tumor growth curves in C57BL/6 mice treated with miR-192-5p antagomir NC, miR-192-5p antagomir, anti-CD25+miR-192-5p antagomir NC, anti-CD25+miR-192-5p antagomir, anti-IL-10 + miR-192-5p antagomir NC, anti-IL-10 + miR-192-5p antagomir. **(B)** Flow cytometry analysis of Tregs in tumor and **(C)** spleen. Data are pooled from six independent experiments. Statistical analysis between two groups was conducted using two-tailed t-test. Error bars, SD. *P < 0.05, **P < 0.01, ****P < 0.0001. n = 5/group.


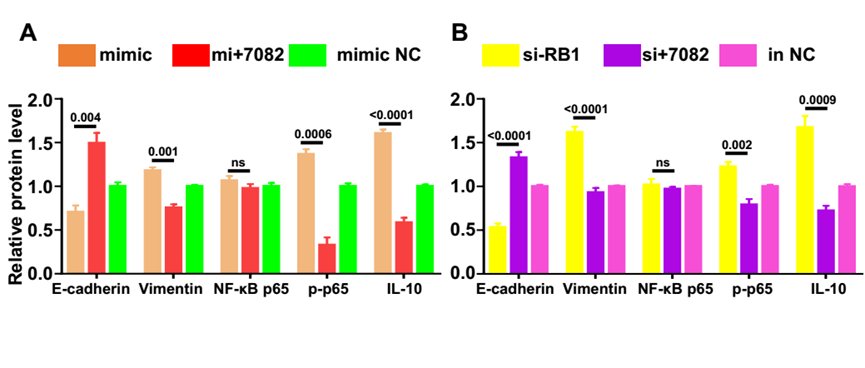


**Figure S12.**WB quantification of protein levels in GC cells. **(A)** Western blot quantification of E-cadherin, Vimentin, NF-κBp65, p-p65 and IL-10 in the BGC-823 cells. BGC-823 cells were transfected with miR-192-5p mimic, mimic NC, BAY11-7082 (10μM). **(B)** Western blot quantification of E-cadherin, Vimentin, NF-κBp65, p-p65 and IL-10 in the BGC-823 cells. BGC-823 cells were transfected with si-RB1, si NC, BAY11-7082 (10μM). Data are pooled from three independent experiments. Statistical analysis between two groups was conducted using two-tailed t-test. One‐way ANOVA statistical tests were adopted for more than two groups. Error bars, SD.

Table S1 Clinicopathologic parameters of gastric cancer patients (n = 30)

| Parameters | n (%) | miR-192-5p expression | miR-192-5p expression | P value |
| --- | --- | --- | --- | --- |
|  |  | High (n=15) | Low (n=15) |  |
| Gender |  |  |  | 1.000 |
| Female | 5 | 3 (10%) | 2 (6.7%) |  |
| Male | 25 | 12 (40%) | 13 (43.3%) |  |
| Age |  |  |  | 0.357 |
| <60 |  | 5 (16.7%) | 8 (26.7%) |  |
| ≥60 |  | 10 (33.3%) | 7 (23.3%) |  |
| T stage |  |  |  | 1.000 |
| T1 | 1 | 1 (3.3%) | 0 (0%) |  |
| T2 | 1 | 0 (0%) | 1 (3.3%) |  |
| T3 | 17 | 8 (26.7%) | 9 (30%) |  |
| T4 | 11 | 6 (20%) | 5 (16.7%) |  |
| N stage |  |  |  | **0.021** |
| N0 | 5 | 0 (0%) | 5 (16.7%) |  |
| N1 | 6 | 2 (6.7%) | 4 (13.3%) |  |
| N2 | 4 | 2 (6.7%) | 2 (6.7%) |  |
| N3 | 15 | 11 (36.7%) | 4 (13.3%) |  |
| Pathologic stage |  |  |  | 0.080 |
| Stage II | 7 | 1 (3.3%) | 6 (20%) |  |
| Stage III | 23 | 14 (46.7%) | 9 (30%) |  |
| Tumor grade |  |  |  |  |
| Well/moderate | 22 | 11 (36.7%) | 11 (36.7%) |  |
| Poor | 8 | 4 (13.3%) | 4 (13.3%) |  |
| recurrence or metastasis |  |  |  | 0.462 |
| 0 | 13 | 5 (16.7%) | 8 (26.7%) |  |
| 1 | 17 | 10 (33.3%) | 7 (23.3%) |  |

Table S2 The clinicopathologic parameters of GC patients and healthy volunteers

|  |  | GC patient | Healthy volunteer | P value |
| --- | --- | --- | --- | --- |
| parameters | n (%) | n=30 | n=40 |  |
| gender |  |  |  | 0.103 |
| female | 15 | 5 (8.3%) | 8 (20%) |  |
| male | 45 | 25 (41.7%) | 32 (80%) |  |
| Age |  |  |  | 0.113 |
| <60 | 24 | 13 (21.7%) | 17 (42.5%) |  |
| ≥60 | 36 | 17 (28.3%) | 23 (57.5%) |  |

Table S3 Univariate and multivariate Cox regression analysis in GC patients (n = 30).

| Characteristics | Univariate analysis | | |  | Multivariate analysis | | |
| --- | --- | --- | --- | --- | --- | --- | --- |
|  | Hazard ratio (95% CI) | P value | |  | Hazard ratio (95% CI) | | P value |
| Age (<60 vs ≥60) | 1.480 (0.608-3.606) | 0.388 | |  |  | |  |
| Gender (Male vs Female) | 0.605 (0.194-1.885) | 0.386 | |  |  | |  |
| T stage (T3-T4 vs T1-T2) | 78667205.183 (0.000-Inf) | 0.998 | |  |  | |  |
| N stage (N2-N3 vs N0-N1) | 1.153 (0.464-2.864) | 0.760 | |  |  | |  |
| TNM Stage^#^ (II vs III) | 0.366 (0.117-1.145) | | 0.084 |  | 0.571 (0.150-2.169) | 0.411 | |
| Tumor grade (Well/ Moderate vs Poor) | 0.755 (0.270-2.113) | | 0.592 |  |  |  | |
| recurrence or metastasis (Yes vs NO) | 4.749 (1.584-14.239) | | **0.005** |  | 3.682 (1.198-11.320) | **0.023** | |
| miR-192-5p | 1.496 (1.175-1.905) | | **0.001** |  | 1.377 (1.033-1.835) | **0.029** | |

Notes: ^#^The 7th edition of the AJCC Cancer Staging Manual. Abbreviations: TNM, tumor-node-metastasis.

Table S4 Expressions of 14 miRNA based on three GEO databases

| miRNA name | GSE 78775 | | | | GSE86226 | | | | GSE164174 | | | |
| --- | --- | --- | --- | --- | --- | --- | --- | --- | --- | --- | --- | --- |
|  | t | logFC  (Tumor vs Normal) | p.value | adj. p.value | t | logFC  (Tumor vs Normal) | p.value | adj. p.value | t | logFC  (Tumor vs Normal) | p.value | adj. p.value |
| hsa-miR-375 | 4.06965 | 2.612735 | 1.52E-04 | 0.009024 | -0.5953 | -0.1899 | 0.582925 | 0.7274 | 8.36272 | 0.823437 | 9.49E-17 | 1.19E-16 |
| hsa-miR-192-5p | 2.30043 | 0.950439 | 2.52E-02 | 0.018247 | 3.3612 | 1.2666 | 0.027214 | 0.116 | 3.97848 | 0.319596 | 7.11E-05 | 7.93E-05 |
| hsa-miR-346 | 2.69314 | 0.561118 | 9.37E-03 | 0.126814 | 1.8082 | 0.9942 | 0.143085 | 0.3174 | 3.86071 | 0.234567 | 1.16E-04 | 1.28E-04 |
| hsa-miR-107 | -2.24738 | -0.206502 | 2.87E-02 | 0.248613 | 0.8386 | 0.2829 | 0.447776 | 0.6412 | 39.93956 | 3.969304 | 4.24E-277 | 1.03E-275 |
| hsa-miR-140-5p | -2.06266 | -0.274652 | 4.39E-02 | 0.321974 | 5.0791 | 1.4893 | 0.006627 | 0.0913 | 14.05451 | 1.360602 | 2.01E-43 | 3.14E-43 |
| hsa-miR-320b | -3.63969 | -0.387326 | 6.05E-04 | 0.021883 | -0.7899 | -0.2975 | 0.472772 | 0.6611  c | 50.073 | 2.539057 | 0 | 0 |
| hsa-miR-320a | -3.13095 | -0.405398 | 2.79E-03 | 0.053658 | -1.8906 | -0.5527 | 0.129895 | 0.2981 | 50.83606 | 1.856656 | 0 | 0 |
| hsa-miR-331-3p | -3.63443 | -0.464494 | 6.15E-04 | 0.021883 | 4.1654 | 1.2053 | 0.013369 | 0.1078 | 4.00343 | 0.332068 | 6.40E-05 | 7.14E-05 |
| hsa-miR-127-3p | -2.80235 | -0.56414 | 7.00E-03 | 0.101868 | -2.4541 | -0.6836 | 0.068578 | 0.2093 | 11.4295 | 1.13546 | 1.31E-29 | 1.84E-29 |
| hsa-miR-125a-5p | -3.51341 | -0.623442 | 8.94E-04 | 0.029628 | 0.0502 | 0.0253 | 0.962343 | 0.9677 | 1.84572 | 0.144402 | 6.50E-02 | 6.85E-02 |
| hsa-miR-324-5p | -4.36053 | -0.682512 | 5.75E-05 | 0.005457 | 1.2865 | 2.2144 | 0.089515 | 0.2356 | 20.72786 | 1.984734 | 5.63E-89 | 1.25E-88 |
| hsa-miR-409-3p | -3.20565 | -0.784351 | 2.25E-03 | 0.046671 | -3.8345 | -2.124 | 0.017704 | 0.1078 | 19.80359 | 1.681527 | 7.45E-82 | 1.55E-81 |
| hsa-miR-199a-5p | -3.1661 | -0.826835 | 2.52E-03 | 0.050492 | 3.7477 | 1.0555 | 0.019108 | 0.1078 | 0.285685 | 3.68778 | 2.30E-04 | 2.54E-04 |

FC, fold change.

| Table S5 The sequences of the primers for qRT-PCR | | |
| --- | --- | --- |
| Genes | Primer Sequence (5' to 3') | Product size (bp) |
| RB1 | F: AGGACCGAGAAGGACCAACTGATC | 182 |
|  | R: CTGGAAGGCTGAGGTTGCTTGTG |  |
| hsa-miR-192-5p | Reverse Transcription (RT) primer: GTCGTATCCAGTGCAGGGTCCGAGGTATTCGCACTGGATACGACGGCTGT |  |
|  | F: GCGCGCTGACCTATGAATTG | 1 |
|  | R: AGTGCAGGGTCCGAGGTATT |  |
| IL-27 | F: GGAGGAGGAGGAGGAGGAGAGG | 110 |
|  | R: GCAGGCGGTAGGTGGAGAGG |  |
| IL-2 | F: GCCCAAGAAGGCCACAGAAC | 126 |
|  | R: TCCCGCTACACTTGTTTTCAC |  |
| TGF-β | F: AAGGACCTCGGCTGGAAGTGC | 136 |
|  | R: CCGGGTTATGCTGGTTGTA |  |
| FOXP3 | F: AAGAACGCCATCCGCCACAAC | 92 |
|  | R: TCCAGCTCATCCACGGTCCAC |  |
| IL-10 | F: GGAAGCTTTTCTACTCAGCAGA | 93 |
|  | R: CACAGATTTTGGCTAGCTCATC |  |
| NF-κB p65 | F: TGTGAAGAAGCGGGACCTGGAG | 114 |
|  | R: AAGCAGAGCCGCACAGCATTC |  |
| IL-23 | F: TTCACAGAAGCTCTGCACACTGG | 128 |
|  | R: GGTCACAGCCATCTCCACACTG |  |
| GAPDH | F: GCACCACCAACTGCTTAGCA | 106 |
|  | R: GTCTTCTGGGTGGCAGTGATG |  |
| U6 | F: CTCGCTTCGGCAGCACA | 96 |
|  | R: AACGCTTCACGAATTTGCGT |  |
| CHIP | F: ATGGTGAGAGCAACATCTAG | 70 |
|  | R: TGCCTGGTTGTCACTTGA |  |
| CHIP NC | F: TAAGAGGAGGAGGTGGGG | 125 |
|  | R: GCATGAGGCGGCCATCCTT |  |

Table S6. Antibodies used in this study

| **Antibodies** | **Source** | **Identifier** |
| --- | --- | --- |
| RB1 | Abcam | Cat# ab181616 |
| RB1 | Cell Signaling Technology | Cat#9309 |
| NF-κB p65 | Abcam | Cat# ab16502 |
| NF-κB p65 | Cell Signaling Technology | Cat#8242 |
| p-NF-κB p65 | Abcam | Cat# ab194726 |
| IL-10 | Proteintech | Cat# 60269-1-Ig |
| E-cadherin | Proteintech | Cat# 60335-1-Ig |
| Vimentin | Proteintech | Cat# 60330-1-Ig |
| PDL1 | Proteintech | Cat# 66248-1-Ig |
| PD-1 | Proteintech | Cat# 66220-1-Ig |
| GAPDH | Proteintech | Cat# 60004-1-Ig |
| STAT3 | Proteintech | Cat# 10253-2-AP |
| p-STAT3 | Bioswamp | Cat# PAB36302-P |
| ERK1/2(137F5) | Cell Signaling Technology | Cat# 4695 |
| p-ERK1/2(Thr202/Tyr204) | Cell Signaling Technology | Cat# 4370 |
| FOXP3 | Abcam | Cat# ab215206 |
| Rabbit mAb IgG | Cell Signaling Technology | Cat#3900 |
| CD4-FITC (Clone OKT4) | Biolegend | Cat# 317407 |
| CD25-APC (Clone BC96) | Biolegend | Cat# 302609 |
| CD25- APC/Cyanine7 (Clone BC96) | Biolegend | Cat# 302613 |
| FOXP3-PE (Clone 206D) | Biolegend | Cat# 320107 |
| PD-1-APC (Clone EH12.2H7) | Biolegend | Cat# 329907 |
| Anti-Argonaute-2 | Abcam | Cat#ab186733 |
| β-actin | Proteintech | Cat#66009-1-Ig |
| Histone-H3 | Proteintech | Cat#17168-1-AP |
| IL-10 neutralizing antibody | PeproTech | Cat# 500-P20-50UG |
| Anti-mouse-IL-10 mAb | Biolegend | Cat# 504908 |
| Anti-mouse-CD25 mAb | BioXcell | Cat# BE0012-1MG |
| FITC anti-mouse CD4 (clone GK1.5) | Biolegend | Cat #100405 |
| APC anti-mouse CD279 (PD-1) (clone 29F.1A12) | Biolegend | Cat #135209 |
| PE anti-mouse/rat/human FOXP3 (clone 150D) | Biolegend | Cat #320007 |
| APC/Cyanine 7 anti-mouse CD25 (clone PC61) | Biolegend | Cat #102025 |

**Materials and Methods**

**Cell culture and transfection**

All cells in this study were maintained in 1640 (Gibco, Carlsbad, CA, USA) supplemented with 10% FBS (Gibco, Carlsbad, CA, USA). The culture environment maintained at 37 °C containing 5% CO2. The miR-192-5p mimic, inhibitor, and their control plasmids (mimic NC, inhibitor NC), siRNA targeted at RB1, p65, and the scrambled negative control (NC) were all purchased from Riobobio (Guangzhou, China). The target sequences: si-RB1-1target sequence: 5’-3’GAACGATTATCCATTCAAA; si-RB1-2 target sequence: 5’-3’GAACGATGTGAACATCGAA; si-RB1-3 target sequence: 5’-3’GAGAGAGCTTGGTTAACTT; si-NF-κB p65-1 target sequence: 5’-3’ GCGACAAGGTGCAGAAAGA; si-NF-κB p65-2 target sequence: CCTGTATACTCTGGAAGTT; si-NC: 5’-3’ UUCUCCGAACGUGUCACGU; 3’-5’ AAGAGGCUUGCACAGUGCA; miR-192-5p mimic: 5’-3’ CUGACCUAUGAAUUGACAGCC; 3’-5’ GACUGGAUACUUAACUGUCGG; miR-192-5p mimic NC: 5’-3’ UUUGUACUACACAAAAGUACUG; 3’-5’ AAACAUGAUGUGUUUUCAUGAC; miR-192-5p inhibitor: 5’-3’ GGCUGUCAAUUCAUAGGUCAG; miR-192-5p inhibitor NC: 5’-3’CAGUACUUUUGUGUAGUACAAA. Gastric cancer cells were transfected with Lipofectamine 2000 (Invitrogen, Carlsbad, CA, USA) according to the manufacturer’s instructions. Silencing efficiency of different siRNAs was evaluated by qRT-PCR.

**Quantitative real-time PCR (qRT-PCR)**

Total RNA from GC tissues, PBMCs and GC cells were isolated using TRIzol reagent (Invitrogen, USA). RNA concentration and purity were measured using a NanoDrop 2000 spectrophotometer (Thermo Fisher Scientific). RNA (1μg) was transcribed into cDNA using PrimeScript RT reagent Kit (Vazyme, China). The qRT-PCR was performed using SYBR Green master mix (Vazyme, China). Relative mRNA and miRNA expression were calculated as 2–ΔΔCt. Primer sequences are listed in Table S5.

**Western blot assay**

Different cells were lysed on ice with RIPA buffer. The proteins were separated by 10% SDS-PAGE and transferred to polyvinylidene difluoride (PVDF) membranes. Then the membranes were blocked with 5% skimmed milk for 2 h and incubated with primary antibodies at 4 °C overnight. The membranes were washed with TBST and then incubated with secondary antibody at room temperature for 1 hour. All antibodies used in Western blot assay were shown as follows: anti-RB1(Abcam, ab181616,1:1000), anti-IL10 (proteintech, 60269-1-Ig,1:1000), anti-NFκB p65(Abcam, ab16502,1:1000), anti-NFκB p-p65(Abcam, ab194726,1:1000), anti-E-cadherin (proteintech, 60335-1-Ig,1:1000), anti-Vimentin (proteintech, 60330-1-Ig,1:1000), GAPDH (proteintech, 60004-1-Ig,1:5000), anti-PDL1 (proteintech, 66248-1-Ig,1:2000), anti-PD-1 (proteintech, 66220-1-Ig,1:2000)

**5-ethynyl-20-deoxyuridine (EdU) cell proliferation assay**

Cells were treated with 10 μM EdU for 2 h. Then the EdU assay was performed with the BeyoClick™ EdU Cell Proliferation Kit (Beyotime, C0071L, China) according to the manufacturer’s instruction. Cell images were captured by using an immunofluorescence microscope.

**Wound healing assay**

Gastric cancer cells were seeded in 6-well plate and grew until ~80% confluence. Then the scratch line was made with sterile pipette tip. After taking pictures at 0h and 48h, Image J software was used for calculating migration rate.

**Transwell migration and invasion assay**

Transwell chamber (8μm pore size; Corning, USA) was used for migration assay. Cell invasion assay was performed with Matrigel-coated Transwell chamber. MKN45 cells and BGC-823 cells were seeded in the upper chamber and incubated for 48h. Then the migrated and invaded cells were fixed in 4% paraformaldehyde and stained with 0.5% crystal violet. Five random fields from each well were counted under a microscope (magnification, ×200).

**Immunofluorescence (IF) assay**

BGC-823 cells and MKN45 cells transfected with different conditions were seeded in 6-well culture plates plated with cell-climbing slices. The cells were fixed with 4% paraformaldehyde, permeabilized in 0.1% Trition X-100/PBS. Then the primary antibodies were added: anti-RB1 (Abcam, ab181616,1:100), anti-NFκB p65 (Abcam, ab16502, 1:100) at 4 °C overnight. Cells were incubated with anti-rabbit FITC-conjugated secondary antibody (FITC, Invitrogen, CA), Alexa-conjugated anti-goat secondary antibody (Alexa647, Invitrogen, CA). Finally, cells were stained with DAPI. The stained cells were observed under fluorescence microscope.

**Immunohistochemistry (IHC) assay**

Paraffin sections of xenograft tumor tissues and GC patient tissues samples were performed on 5-μm sections. The sections were incubated with primary antibodies at 4 °C overnight, then the sections were incubated with secondary antibodies after washing with PBS. All antibodies used in IHC were shown as follows: anti-IL10 (proteintech, 60269-1-Ig,1:100), anti-RB1 (Abcam, ab181616,1:200), anti-FOXP3 (Abcam, ab215206,1:200).
